# Coordination Properties of the Zinc Domains of BigR4 and SmtB Proteins in Nickel Systems—Designation of Key Donors

**DOI:** 10.1021/acs.inorgchem.2c00319

**Published:** 2022-06-13

**Authors:** Anna Rola, Paulina Potok, Robert Wieczorek, Magdalena Mos, Elżbieta Gumienna-Kontecka, Sławomir Potocki

**Affiliations:** † Faculty of Chemistry, University of Wroclaw, 14 Joliot-Curie Street, Wroclaw 50-383, Poland; ‡ WMG, International Manufacturing Centre, University of Warwick, Coventry CV4 7AL, U.K.

## Abstract

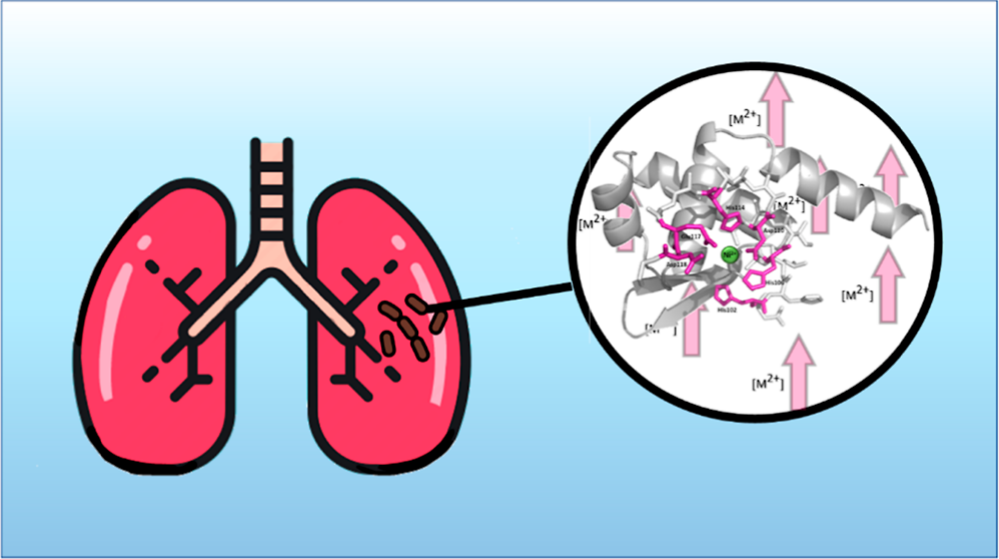

The increasing number of antibiotic-resistant pathogens has become one of the foremost health problems of modern times. One of the most lethal and multidrug-resistant bacteria is *Mycobacterium tuberculosis* (Mtb), which causes tuberculosis (TB). TB continues to engulf health systems due to the significant development of bacterial multidrug-resistant strains. Mammalian immune system response to mycobacterial infection includes, but is not limited to, increasing the concentration of zinc(II) and other divalent metal ions in phagosome vesicles up to toxic levels. Metal ions are necessary for the survival and virulence of bacteria but can be highly toxic to organisms if their concentrations are not strictly controlled. Therefore, understanding the mechanisms of how bacteria use metal ions to maintain their optimum concentrations and survive under lethal environmental conditions is essential. The mycobacterial SmtB protein, one of the metal-dependent transcription regulators of the ArsR/SmtB family, dissociates from DNA in the presence of high concentrations of metals, activating the expression of metal efflux proteins. In this work, we explore the properties of α5 metal-binding domains of SmtB/BigR4 proteins (the latter being the SmtB homolog from nonpathogenic *Mycobacterium smegmatis*), and two mutants of BigR4 as ligands for nickel(II) ions. The study focuses on the specificity of metal–ligand interactions and describes the effect of mutations on the coordination properties of the studied systems. The results of this research reveal that the Ni(II)-BigR4 α5 species are more stable than the Ni(II)-SmtB α5 complexes. His mutations, exchanging one of the histidines for alanine, cause a decrease in the stability of Ni(II) complexes. Surprisingly, the lack of His102 resulted also in increased involvement of acidic amino acids in the coordination. The results of this study may help to understand the role of critical mycobacterial virulence factor—SmtB in metal homeostasis. Although SmtB prefers Zn(II) binding, it may also bind metal ions that prefer other coordination modes, for example, Ni(II). We characterized the properties of such complexes in order to understand the nature of mycobacterial SmtB when acting as a ligand for metal ions, given that nickel and zinc ArsR family proteins possess analogous metal-binding motifs. This may provide an introduction to the design of a new antimicrobial strategy against the pathogenic bacterium *M. tuberculosis*.

## Abstract 2

The histidine-rich α5 domains of SmtB (L4, *Mycobacterium tuberculosis*) and BigR4 (L1, *Mycobacterium smegmatis*) were studied as ligands for Ni(II). Point mutation analysis of L1 revealed that His102 and 106 preferably bind metal ions. The general Ni(II)-binding motif for both of the ligands was established as HX3HX3DX3HX2ED. L1 forms more stable complexes than L4 due to the stabilizing effect of arginine residues.

## Introduction

*Mycobacterium tuberculosis* is one of the pathogenic species of the *Mycobacterium* family, responsible for the dangerous human disease known as tuberculosis (TB).^1−3^ The latest statistics collected by the World Health Organisation (WHO) show that in 2020 1.3 million people died due to TB and an estimated 9.9 million people fell ill with the disease worldwide.^4^ The issue is more problematic when considering the rapid development of TB resistance to commonly used antibiotics, so-called multidrug-resistant TB (MDR-TB).^5−8^ WHO estimates that 484k patients show resistance to rifampicin, one of the most effective first-line antiTB drugs.^9−11^ Metal-based antimicrobial therapy could be a promising TB treatment strategy. Interestingly, *M. tuberculosis* can survive in an organism by penetrating the host macrophages and manipulating its metal cation trafficking, while the mammalian immune system defends itself from *M. tuberculosis* infection by increasing copper and zinc concentrations in phagosomes.^12^ Therefore, in order to develop a new method of treatment it is necessary to understand the mechanisms of metal homeostasis in *M. tuberculosis*. One such mechanism is controlled by helix-turn-helix (HTH) -type ArsR-SmtB family proteins—significant in the virulence process. They are capable of sensing a wide variety of metal ions such as Zn (SmtB Synechococcus sp., UniProtKB-P30340), Cd and Pb (CmtR, *M. tuberculosis*, UniProtKB-P9WMI9), and Ni and Co (NmtR, KmtR *M. tuberculosis*, UniProtKB-069711 and 053838). They possess one or both of two structurally distinct metal coordination sites: a cysteine-rich α3N site that forms S_3_ or S_4_ complexes with large, soft metal ions including Cd(II), Pb(II), and Bi(III), and the second, an α5 site comprised of carboxylate and imidazole ligands which preferentially binds smaller and harder metal ions such as Zn(II), and Ni(II).^13,14^

**Figure 1:**
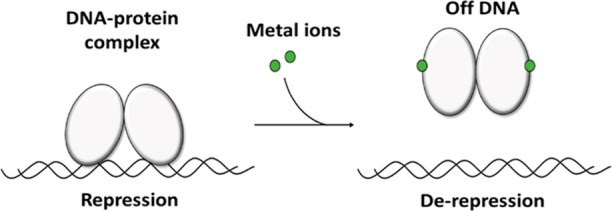
Model of the mechanism of gene regulation in the ArsR-SmtB family. Based on [15].

The role of ArsR family proteins is to repress the expression of genes and operons related to stress-inducing concentrations of various heavy metal ions.^15^ Although the exact regulation mechanism of the metal ions intracellular bioavailability by ArsR-SmtB has not been precisely described, there are a few facts known already. When direct metal ion binding occurs, a complex of DNA-protein dissociates, and ArsR-SmtB controlled gene/operon begins to express zinc export proteins (Figure 1).^16^

**Figure 2:**
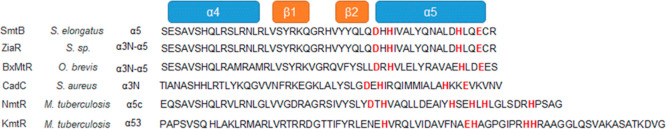
Fragments of some of the ArsR-SmtB family repressors sequences. Red letters represent metal-binding sites. Based on[15].

*M. tuberculosis* regulates its zinc homeostasis with the use of SmtB protein (UniProtKB-P9WMI5).^17^ Recently, we described a zinc coordination model peptide of mycobacterial SmtB α5 domain,^18^ and compared it to the α5 site of the well-described *Synechococcus elongatus* SmtB (*S. elongatus*, UniProtKB-P30340). The sequence identity of metal-binding domains from both proteins is 30% (Table 1). Based on the sequence identity, we recognized the general motif of the α5 domain for compared proteins: DXHXAXXXXXAXXHXXE(H) (Figure 2). It applies to another ArsR-SmtB family protein—Ni(II) sensing NmtR (*M. tuberculosis*, UniProtKB-069711). Sequence similarity of SmtB and NmtR (*M. tuberculosis*) also equals 30%. NmtR is responsible for the maintenance of nickel ion homeostasis in *M. tuberculosis*.^19^ According to the literature, NmtR and SmtB possess analogous metal-binding motifs.^15^ In this paper we characterize the SmtB α5 domain as a ligand for Ni(II) ions from the coordination and bioinorganic chemistry points of view. Knowledge of metal-complex parameters such as geometry, and stability are significant in drug-resistant bacteria research.^20−22^ Moreover it may help us understand the overall mechanism of *M. tuberculosis* homeostasis.

**Table 1:** Comparison of Metal Sensing Domains of SmtB from *S. elongatus* and *M. tuberculosis* with NmtR Metal Sensing Domain from *M. tuberculosis*. Results of UniProt

SmtB metal sensing domains from *M. tuberculosis* (UniProtKB-P9WMI5)	SmtB metal sensing domains from *S. elongatus* (UniProtKB-P30340)	NmtR metal sensing domains from *M. tuberculosis* (UniProtKB-069711)
_116_DHHLAHIVLDAVAHAGE_132_	_104_DHHIVALYQNALDHLQE_120_	_91_DTHVAQLLDEAIYHSEH_107_
DXHXAXXXXXAXXHXXE	DXHXAXXXXXAXXHXXE	DXHXAXXXXXAXXHXXH

Zn(II) is a spectroscopically silent metal ion; it is d^10^ that hampers UV–vis spectra and does not possess any isotope which would enable its characteristics by NMR.^23^ This problem can be solved by replacing Zn(II) with other metal ions with appropriate spectroscopic parameters. Despite different preferences of coordination mode, there is a similarity between Ni(II) and Zn(II). Both of them belong to a group of borderline acids in the Brønsted–Lowry theory of acids and bases. They are able to bind the same type of ligands; for instance imidazole ring of histidine.^13,24^

SmtB from *S. elongatus* preferably coordinates with zinc ions, it is also able to sense other metal ions, for example, Cd(II) and Cu(II), with which it can form complexes with tetrahedral geometry.^25^ Interestingly, SmtB may also bind metal ions, which prefer other coordination modes, for example, Ni(II) or Hg(II) with variable affinities.^25,26^ Recent studies have shown that noncobalt sensing SmtB has a high affinity for cobalt, and nonzinc sensing NmtR has a high affinity to zinc.^27,28^ For these abovementioned reasons we decided to focus on the properties of the SmtB α5 domain as a ligand for Ni(II) ions. We believe that to fully characterize the metal-binding α5 domain, it is necessary to consider the reported strong possibility of its interaction with other metal ions than Zn(II).

Another interesting HTH-ArsR transcriptional regulator in which the DXHXAXXXXXAXXHXXE motif occurs is BigR4 from *Mycobacterium smegmatis*.^29^ This particular species of the *Mycobacterium* family is nonpathogenic making it an excellent model organism for *M. tuberculosis* behavior studies.^30−32^ The interaction between the BigR4 binding domain and metals has not been characterized well. In order to describe the interactions between the metal-binding domain of BigR4 and SmtB from *M. tuberculosis* with Ni(II), we used a variety of complementary analytical methods. In this work, we examined model peptides originating from SmtB (Ac-_116_DHHLAHIVLDAVAHAGEDAI_135_) and BigR4 (Ac-_101_DHHLAHIVVDAIAHASEDRR_120_) containing DXHXAXXXXXAXXHXXE motif in order to characterize Ni(II) binding sites. The geometry of the formed complexes as well as their thermodynamic properties, such as stability constants, were also characterized. We continued our research with the two of the BigR4 mutants (Ac-_101_DAHLAHIVVDAIAHASEDRR_120_ and Ac-_101_DHHLAAIVVDAIAHASEDRR_120_) in which one of the histidine residues is replaced with an alanine residue. We chose these specific mutant peptides for two main reasons: (i) histidines are one of the most potent Ni(II)-interacting donors, and more importantly, (ii) these particular histidine residues are not likely to be involved in direct interactions with zinc in this and similar protein families,^15^ but it could bind Ni(II)—a metal of coordination number = 6. We hope that our studies may show which His is directly involved in the Ni(II)-binding. The graphical representation of the model peptides is shown in Table 2.

**Table 2:**
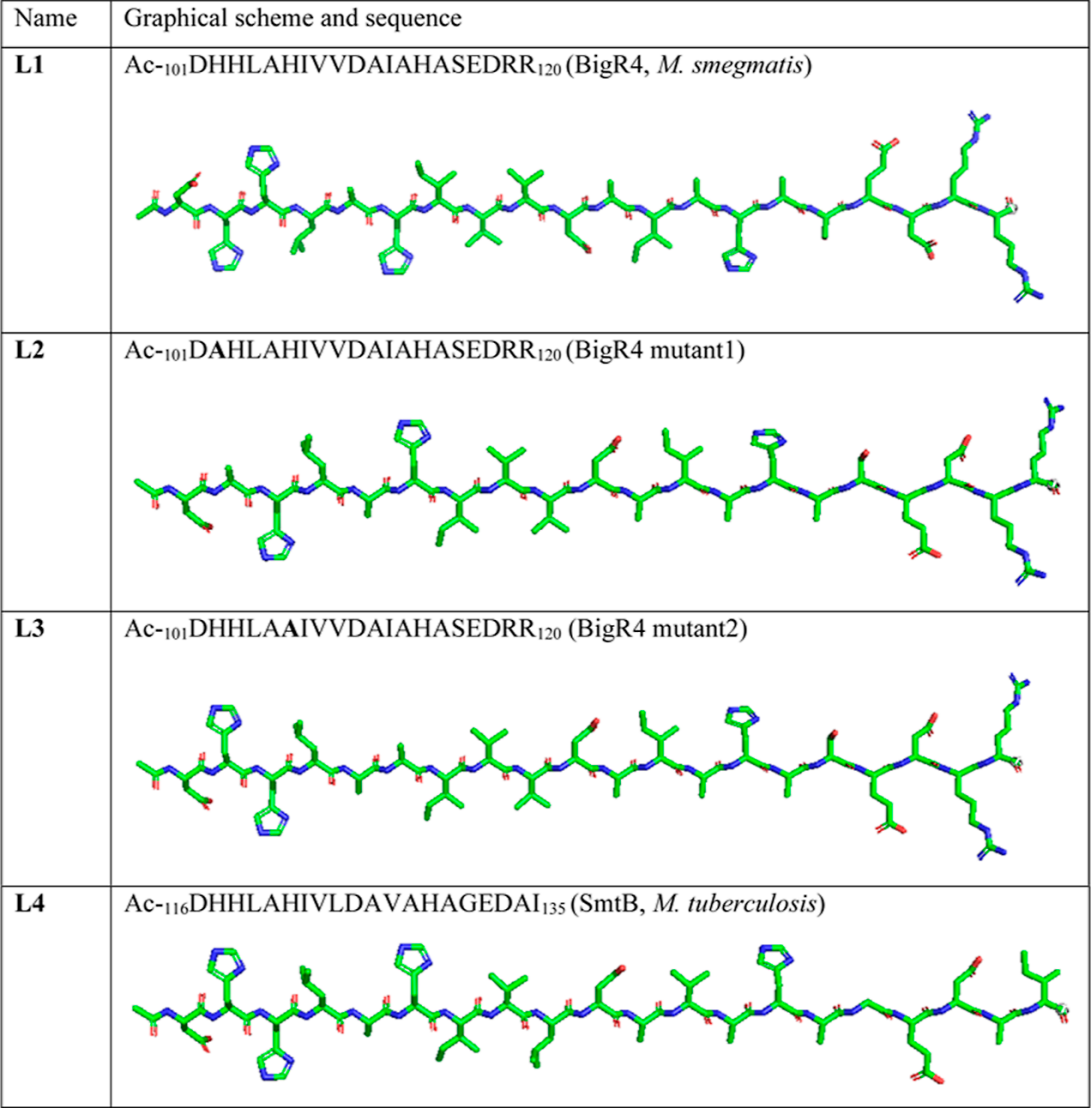
Graphical Representation of the Model Peptides

Results of our research reveal interesting details regarding the coordination chemistry of the α5 domain of SmtB (*M. tuberculosis*) and BigR4 (*M. smegmatis*), strongly encouraging further investigation of interactions between metal ions and particular histidine-rich domains of proteins engaged in the virulence process.

## Experimental Section

### I Materials

All peptide ligands were purchased from KareBay Biochem, Inc. (certified purity: L1 = 98.27%, L2 = 98.08, L3 = 98.46, and L4 = 98.25%). The identity of peptides was evaluated based on mass spectrometry. The purity was evaluated based on potentiometric titrations using the Gran method.^33^ The solutions of metal ions were prepared using NiCl_2_ (POCh, HPLC grade). The metal salt was dissolved in filtered double-distilled water. The concentration of a stock solution was periodically checked via ICP. All sample solutions were prepared with freshly double-distilled water. For the preparation of solutions of the peptide, 4 × 10^–3^ M HCl (Merck) acid was used. The ionic strength was adjusted to 0.1 M by adding KCl (Merck).

### II Mass Spectrometry Measurements

Electrospray ionization-mass spectrometry (ESI-MS) experiments were performed on the LCMS-9030 qTOF Shimadzu (Shimadzu, Kyoto, Japan) device equipped with a standard ESI source and the Nexera X2 system. Analysis was performed in the positive ion mode, between 100 and 3000 *m*/*z*. LCMS-9030 parameters: nebulizing gas–nitrogen, nebulizing gas flow 3.0 L/min, drying gas flow—10 L/min, heating gas flow—10 L/min, interface temperature 300 °C, desolvation line temperature −400 °C, detector voltage −2.02 kV, interface voltage 4.0 kV, collision gas–argon, mobile phase (A) H2O +0.1% HCOOH and (B) MeCN +0.1% HCOOH, and mobile phase total flow −0.3 mL/min. The injection volume was optimized depending on the intensity of the signals observed on the mass spectrum within the range of 0.1–1 μL. Obtained signals had a mass accuracy error in the range of 1 ppm. The concentration of peptide was 0.1 mM, and M/L molar ratio was 1:1. Samples were prepared in a mixture of water/methanol (50/50 v/v) at pH 7.40. All used solvents were of LC–MS grade. The obtained data were analyzed by LabSolutions software (Shimadzu, Kyoto, Japan).

### III Potentiometric Measurements

Stability constants for the proton as well as Ni(II) complexes were calculated from the pH-metric titration curves obtained under an argon atmosphere (in order to provide the sample from carbonates appearance) in the pH range 2.5–11 at 298 K and ionic strength of 0.1 M KCl. The measurements were performed using a Dosimat 665 Methrom titrator connected to a Methrom 691 pH-meter equipped with a pH electrode InLab Semi-Micro (Mettler Toledo). The thermostabilized glass cell was equipped with a magnetic stirring system, a microburette delivery tube, and an inlet-outlet tube for argon. Solutions were titrated with 0.1 M carbonate-free NaOH. The electrodes were calibrated daily for hydrogen ion concentration by titrating HCl with KOH under the same experimental conditions as mentioned above. The Gran method allowed us to establish the purities and the exact concentrations of the ligands solutions.^33^ The ligand concentration was 0.5 mM, and the Ni(II) to ligand molar ratio was 1:1.

Stability constant calculations were performed using HYPERQUAD 2006 program.^34^ Reported log β values refer to the overall equilibria
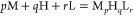
1
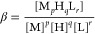
2where log K_step_ values refer to the protonation process (charges are omitted for clarity).
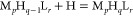
3(charges omitted; p might also be 0). Standard deviations were calculated by HYPERQUAD 2006 and refer to random errors only. For Ni(II) complexes, the minimum waiting time before adding each drop of the base was 60 s. The *K*_d_ values, speciation, and competition diagrams were computed with the HYSS program.^35^

### IV NMR Measurements

NMR spectra were recorded at 14.1 T on a Bruker Avance III 600 MHz equipped with a Silicon Graphics workstation. The temperatures were controlled with an accuracy of ±0.1 K. Suppression of the residual water signal was achieved by excitation sculpting, using a selective square pulse on water 2 ms long. All the samples were prepared in a 90% H_2_O and 10% D_2_O (99.95% from Merck) mixture. Proton resonance assignment was accomplished by 2D ^1^H–^1^H total correlation spectroscopy and nuclear Overhauser effect spectroscopy experiments, carried out with standard pulse sequences. Spectral processing and analysis were performed using Bruker TOPSPIN 2.1, Cara, and Sparky. Samples of analyzed complexes were prepared by adding metal ions to an acidic solution of 0.8 mM ligand (pH 5.2), and the pH was then increased to a higher value (pH 7.4).

### V CD and UV–Vis Spectroscopy Measurements

The absorption spectra were recorded on a Cary 300 Bio spectrophotometer, and the circular dichroism (CD) spectra were recorded on a Jasco J-1500 spectropolarimeter in the 240–800 nm range, using a quartz cuvette with an optical path of 1 cm in the visible and near-UV range and 0.01 cm in the spectral range of 180–260 nm at 298 K. The parameters of the instrument were as followed: scanning speed: 500 nm/min, data pitch: 0.5 nm, and number of accumulations: 3. The concentration of the ligands was 4 × 10^–4^ M for the complexes. Molar ratios of metal to ligand were 1:1. Data were processed using Origin 9.0.

### VI DFT Calculations

Computational methods of theoretical chemistry have been used as a useful tool to predict the structure and stability of the ligands and complexes.^36−38^ Molecular orbital studies on Ni(II) cations’ 1:1 complexes with Ac-_116_DHHLAHIVLDAVAHAGEDAI_135_ and Ac-_101_DHHLAHIVVDAIAHASEDRR_120_ ligands have been done on the density-functional theory (DFT) level of theory with IEFPCM solvent (water) model introduced upon potential energy surface investigation. The starting structure of the peptide for DFT calculations was generated on the basis of the amino acid sequence after 75 ps simulation at 300 K, without cut-offs using BIO+ implementation of CHARMM force field. DFT calculations were performed with Gaussian 09 C.01 suite of programs using the ωB97X-D long-range corrected hybrid density functional with damped atom–atom dispersion corrections used with double-ζ 6-31G(d,p) basis set. All presented structures were fully optimized, and all presented complexes are thermodynamically stable.

## Results and Discussion

Properties of 4 ligands and their Ni(II) complexes such as stability, geometry, binding sites, and stoichiometry were studied by a variety of methods. The sequences of examined ligands were as follows: L1 Ac-_116_DHHLAHIVVDAIAHASEDRR_135_ (original sequence of BigR4 postulated as the metal-binding domain from *M. smegmatis*), L2 Ac-_101_DAHLAHIVVDAIAHASEDRR_120_ (first mutant of the BigR4-binding domain), L3 Ac-_101_DHHLAAIVVDAIAHASEDRR_120_ (second mutant of BigR4-binding domain), and L4 Ac-_116_DHHLAHIVLDAVAHAGEDAI_135_ (original sequence of SmtB metal-binding domain of *M. tuberculosis*).

### Protonation of the Ligands

Protonation constants of examined peptides and probable assignments to the particular chemical groups are presented in Table 3. Charges of the species have been omitted in order to keep the transparency of the table. The peptides were protected at the N-termini, while C-terminal carboxyl groups remained free (C-terminus of the protein). Protonation constants of the ligands have been assigned to the protonation of the side chain groups as well as the C-terminal carboxyl group. Ac-_101_DHHLAHIVVDAIAHASEDRR_120_ peptide (L1) and Ac-_116_DHHLAHIVLDAVAHAGEDAI_135_ (L4) at pH range 2–11 behave like H_9_L acids, while L1 mutants: Ac-_101_DAHLAHIVVDAIAHASEDRR_120_ (L2) and Ac-_101_DHHLAAIVVDAIAHASEDRR_120_ (L3) exhibit eight protonation constants.

**Table 3:** Potentiometric Data for Proton and Ni(II) Complexes of Ac-_101_DHHLAHIVVDAIAHASEDRR_120_ (L1), Ac-_101_DAHLAHIVVDAIAHASEDRR_120_ (L2), Ac-_101_DHHLAHIVVDAIAAASEDRR_120_ (L3), and Ac-_116_DHHLAHIVLDAVAHAGEDAI_135_ (L4) Peptides and Potential Assignments to Appropriate Side Chains/Chemical Groups^a^

	Ac-DHHLAHIVV DAIAHASEDRR-OH (L1)		Ac-DAHLAHIVV DAIAHASEDRR-OH (L2)		Ac-DHHLAAIVV DAIAHASEDRR-OH (L3)		Ac-DHHLAHIVL DAVAHAGEDAI-OH (L4)	
species	log β	p*K*a	log β	p*K*a	log β	p*K*a	log β	p*K*a
HL	9.54 (4)	9.54 (His)	9.65 (2)	9.65 (His)	9.45 (3)	9,45 (His)	9.63 (2)	9.63 (His)
H_2_L	16.98 (6)	7.44 (His)	16.84 (4)	7.19 (His)	16.38 (5)	6,93 (His)	16.90 (5)	7.27 (His)
H_3_L	23.72 (6)	6.74 (His)	23.51 (3)	6.67 (His)	23.10 (4)	6,71 (His)	23.71 (6)	6.80 (His)
H_4_L	30.07 (6)	6.35 (His)	29.61 (3)	6.10 (Glu)	29.09 (4)	5,91 (Glu)	29.97 (5)	6.26 (His)
H_5_L	35.87 (6)	5.80 (Glu)	34.30 (4)	4.69 (Asp)	33.54 (5)	4,55 (Asp)	35.71 (3)	5.74 (Glu)
H_6_L	40.35 (7)	4.49 (Asp)	38.45 (4)	4.15 (Asp)	37.56 (5)	4,02 (Asp)	40.32 (7)	4.62 (Asp)
H_7_L	44.34 (8)	3.98 (Asp)	41.94 (4)	3.49 (Asp)	40.98 (5)	3,42 (Asp)	44.40 (7)	4.08 (Asp)
H_8_L	47.68 (9)	3.34 (Asp)	45.29 (3)	3.35(COOH)	43.55 (5)	2,97(COOH)	48.02 (8)	3.62 (Asp)
H_9_L	50.91 (9)	3.23 (COOH)					51.19 (8)	3.16 (COOH)
Ni(II) Complexes								
NiH_4_L	34.10 (4)		32.59 (2)				33.27(13)	
NiH_3_L			26.98 (3)	5.61 (Glu)	26.60 (3)		27.70 (8)	5.58 (His)
NiH_2_L	22.08 (3)		21,00 (2)	5.98 (His)			21.64 (6)	6.06 (His)
NiHL	15.38 (3)	6.70 (His)	14.69 (1)	6.31 (His)	14.28 (2)		15.18 (5)	6.46 (His)
NiL			5.24 (3)	9.45 (His)	6.00 (2)	8.28 (His)		
NiH_-1_L	–2.73 (4)				–2.75 (1)	8.75 (amide)	–2.58 (5)	
NiH_-2_L			–13.49 (1)		–11.69 (2)	8.93 (amide)		
NiH_-3_L	–22.40 (4)		–22.99 (2)	9.50 (amide)	–21.47 (3)	9.78 (amide)	–22.20 (6)	

a The ligand concentrations were 0.001 M. The Ni(II) to ligand molar ratios were 1:1. I = 0.1 M KCl and *T* = 298 K.

### Protonation Equilibria of the Model Peptides (L1-L4)

Potentiometric measurements detected nine constants (H_9_L) for the Ac-_101_DHHLAHIVVDAIAHASEDRR_120_ (L1) and Ac-_116_DHHLAHIVLDAVAHAGEDAI_135_ (L4) peptides. The exact p*K*_a_ values of both ligands are listed in Table 3. The first constant comes from the C-terminus deprotonation. The next three p*K*_a_ values arise from the deprotonation of three carboxylic side chain groups of aspartic acid residues, while the following one corresponds to the deprotonation of the glutamic acid side chain group. The last four p*K*_a_ values arise from the deprotonation of four histidine imidazole groups.

The Ac-_101_DAHLAHIVVDAIAHASEDRR_120_ (L2) and the Ac-_101_DHHLAAIVVDAIAHASEDRR_120_ (L3) peptides can be considered as H_8_L ligands. The first p*K*_a_ value can be assigned to the deprotonation of the C-terminus. The next three p*K*_a_ values arise from the deprotonation of the carboxylic side chain groups of three aspartic acid residues, the following one is the result of the deprotonation of the glutamic acid side chain group, and the last three constants are related to the histidine imidazole groups (Table 3).

### Metal Complexes

**Figure 3:**
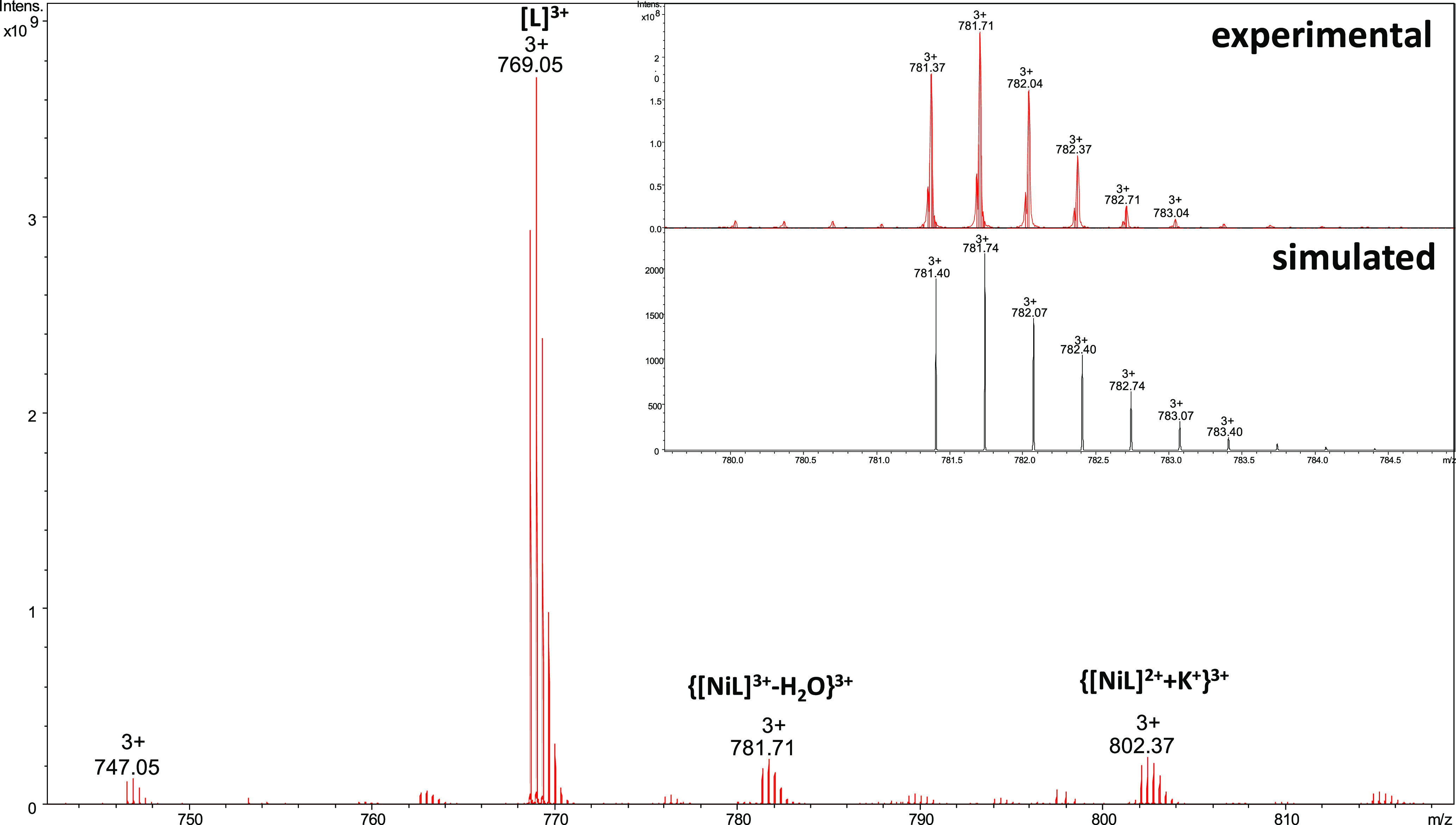
ESI-MS spectrum of the metal-ligand system composed of Ac-_101_DHHLAHIVVDAIAHASEDRR_120_ (L) and nickel(II) ions in the *m*/*z* 740–820 range at pH 7.4 [M/L = 1:1]. The simulated and experimental isotopic distribution spectra of the peak at *m*/*z* = 781.71 are presented in the right corner.

**Figure 4:**
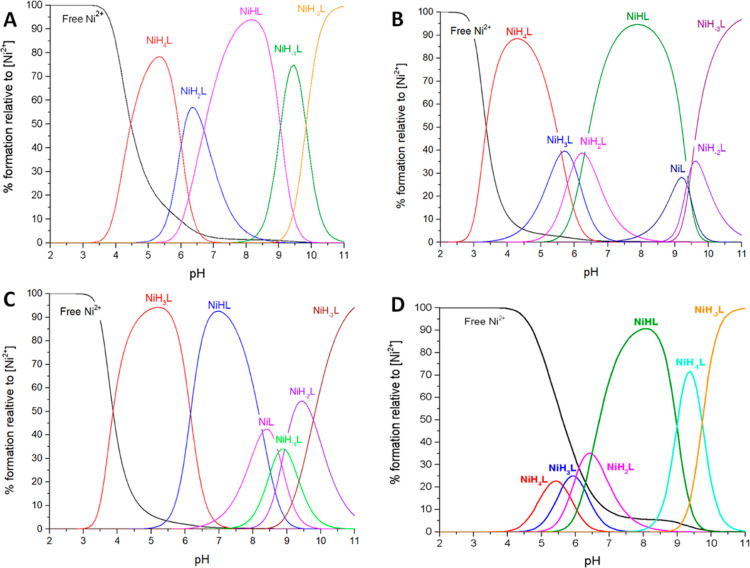
Distribution diagram of complex forms in the Ni(II)-L systems. L—(A) Ac-_101_DHHLAHIVVDAIAHASEDRR_120,_ (B) Ac- _101_DAHLAHIVVDAIAHASEDRR_120_, (C) Ac-_101_DHHLAAIVVDAIAHASEDRR_120_, and (D) Ac-_116_DHHLAHIVLDAVAHAGEDAI_135_. M/L = 1:1; pH range 2–11.

**Figure 5:**
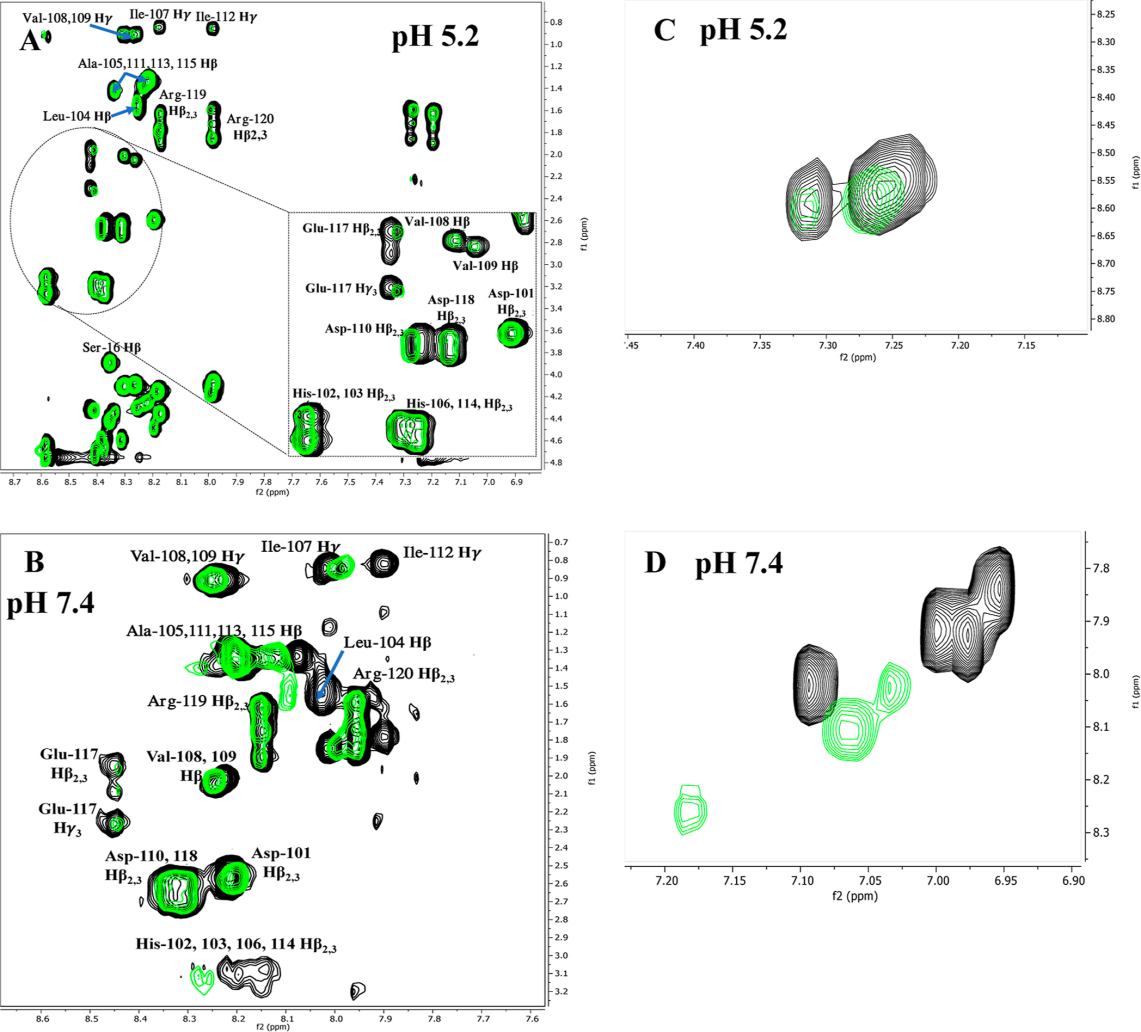
^1^H–^1^H TOCSY NMR spectra of a fragment of the ligand (black) and the Ni(II) complex (green) with the ligand Ac-_101_DHHLAHIVVDAIAHASEDRR_120_ at pH 5.2 (A, C) and at pH 7.4 (B, D); finger print region—left and aromatic region—right; M/L = 0.4:1 and *T* = 298 K.

**Figure 6:**
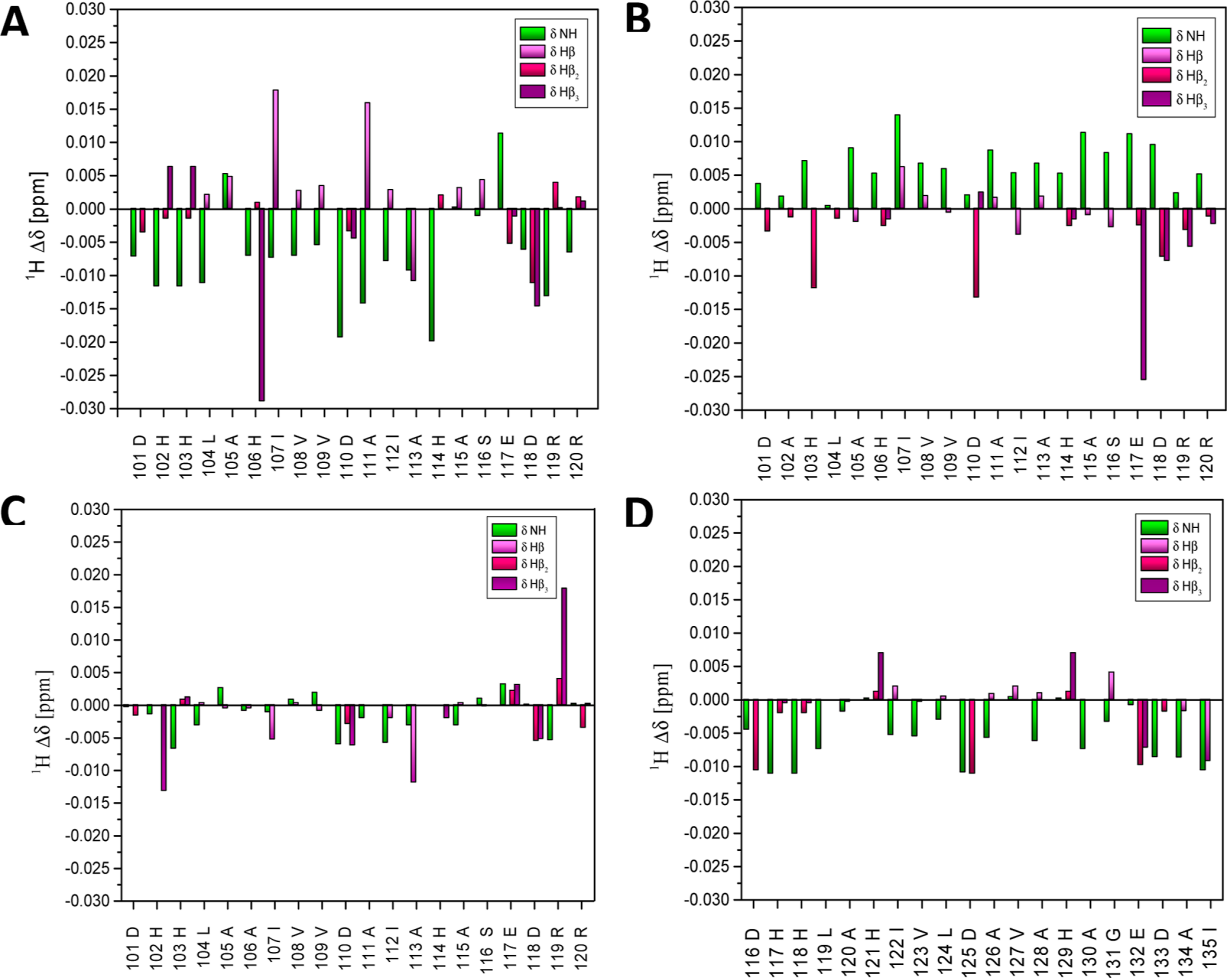
Chemical shift variation induced by Ni(II) at pH 5.2 on (A) L1, (B) L2, (C) L3, and (D) L4 protons.

**Figure 7:**
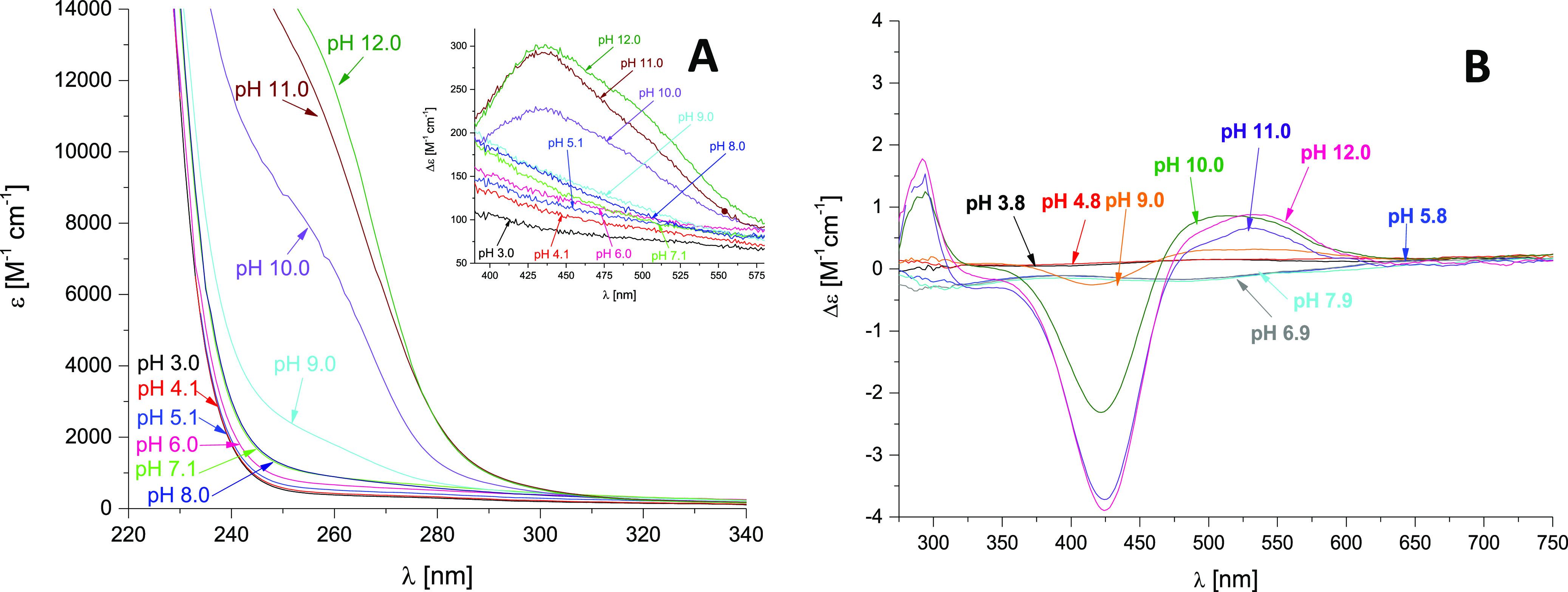
UV–vis (A) and CD (B) spectra of Ni(II) complexes with Ac-_101_DHHLAHIVVDAIAHASEDRR_120_ L1 peptide over the pH range 2–11. Conditions: *T* = 298 K and metal to ligand ratio = 1:1; [Ni(II)] = 2.5 × 10^–4^ M.

The presence of Ni(II) complexes with examined peptides was confirmed by a variety of methods. Signals in the mass spectra have been assigned to ions of ligands or Ni(II)-L complexes. ESI-MS peak assignments were based on the comparison between the precise calculated and experimental *m*/*z* values and their isotopic patterns. To establish metal-binding sites as well as geometry and stability of the complexes, potentiometric titrations, CD, and UV–vis and NMR spectroscopy have been used. Potentiometric data for examined complexes are collected in Table 3; the MS/NMR/UV–vis/CD spectra are presented in Figures 3–7 and Supporting Information

### Ni(II) Complexes of the Ac-_101_DHHLAHIVVDAIAHASEDRR_120_ (L1)

Mass spectra analysis revealed that ligand Ac-_101_DHHLAHIVVDAIAHASEDRR_120_ (L1) forms only equimolar complexes with Ni(II) (Figure 3). Signal *m*/*z* = 781.71 {[NiL]^3+^-H_2_O}^3+^ corresponds to the ion of the complex after the neutral loss of the water molecule. We can also observe the presence of signals corresponding to free ligand L1 ([L]^3+^*m*/*z* = 769.05) and the adduct of the complex with the K^+^ ion ({[NiL]^2+^+K^+^}^3+^*m*/*z* = 802.37).

According to the calculations based on potentiometric data, 5 forms of Ni(II)-L1 were detected. The first observed species is NiH_4_L (Table 3 and Figure 4A) which is formed after deprotonation of the C-terminus and four acidic residues. It starts at pH 4, reaching a maximum at pH 5. The first spectroscopically detectable Ni(II) complex form observed at acidic pH is NiH_2_L, formed after deprotonation of two histidine residues. It starts at pH 5 and reaches a maximum at around pH 6.

The formation of appropriate complex species was confirmed by NMR analysis. After the addition of 0.4 equiv of Ni(II) ions to L1 at pH 5.2, selective chemical shift variations were detected by comparing H^1^–H^1^ TOCSY spectra recorded for apo- and Ni(II)-bound forms (Figure 5A).

The quantitative analysis of chemical shift variations in the NMR spectra collected at pH 5.2 (Figure 6A) shows the largest shift of correlation signals of NH-Hβ protons of His106, Asp110, His114, Glu117, and Asp118. Additionally, the large chemical shift variations observed on Glu117 Hγ proton (Figure 5A) as well as partially on His aromatic protons (Figure 5C) strongly confirm the involvement in the coordination sphere of glutamic acid and two histidine imidazole nitrogen.

The UV–vis and CD spectra recorded at pH range 3.0–8.0 (Figure 7A,B), do not show relevant changes, thus suggesting that the octahedral geometry is maintained.^39^

The next detected species is NiHL. It is formed as a result of the third histidine residue deprotonation. p*K*_a_ value of 6.70 is significantly reduced compared to p*K*_a_ = 7.44 for this residue in the free ligand, suggesting that this histidine is involved in Ni(II) binding (Table 3). In the NMR spectra, the addition of Ni(II) ions at pH 7.4 caused a significant increase in the chemical shifts of HN-Hβ and Hδ-Hε correlations of histidine residues which suggests the exchange of individual acidic amino acids with the remaining histidine in the coordination sphere of the nickel ion (Figures 5B,D and S1A). However, the signals are overlapped, making it impossible to conclude how many histidines bind nickel precisely based only on NMR spectra. The NiH_-1_L species is formed when the fourth histidine, as well as one amide nitrogen, deprotonate. It starts at pH 8 and reaches a maximum at 9.5. In the CD spectrum one can see a band at ca. 280 nm, which can be ascribed to N_am_^–^ → Ni(II) charge transfer transitions.^40,41^ The spectroscopic parameters are consistent with a 4N {1N_am_^–^, 3N_im_} donor set; there is a weak d–d band at the UV–vis spectra with a maximum at 430 nm and d–d bands at 425 and 550 nm in the CD spectra which confirm a square planar geometry of the NiH_-1_L species.^42^ Above pH 9.5, UV–vis band with a maximum at 430 nm and CD bands with max at 425 and 550 nm are still rising which proves that other N_am_^–^ amide nitrogen is getting involved in metal ion binding; the NiH_-3_L complex with a 4N {3N_am_^–^, 1N_im_} donor set is formed, and dominates at pH 10–11.

### Ni(II) Complex of the Ac-_101_DAHLAHIVVDAIAHASEDRR_120_ (L2)

Data obtained in MS experiments revealed that a single molecule of peptide Ac-_101_DAHLAHIVVDAIAHASEDRR_120_ (L2) binds one Ni(II) ion (Figure S2), as reflected by the {[NiL]^3+^-H_2_O}^3+^ signal (*m*/*z* = 759.71) corresponding to the ion of the complex after the neutral loss of water molecule; signals corresponding to the free ligand L2 [L]^3+^ (*m*/*z* = 747.04) and the complex adduct with the K^+^ ion {[NiL]^2+^+K^+^}^3+^ (*m*/*z* = 780.36) were also detected.

The L2 mutant forms seven protonated Ni(II) complexes. The first—NiH_4_L starts below pH 3 and reaches a maximum at around pH 4 (Table 3 and Figure 4B). Probably three acidic side chains of aspartic acids and C-terminal are deprotonated and involved in binding. The next complex, NiH_3_L, reaching maximum at pH 5.5 is characterized by a p*K*_a_ value of 5.61 which is similar to the p*K*_a_ of glutamic acid of free ligand, 6.10 (Table 3), suggesting that this residue does not take part in complexation. The NiH_2_L complex starts to appear at pH 5 and reaches its maximum at pH 6.20. This complex is related to the deprotonation and coordination of the most acidic histidine residue (p*K*_a_ value 5.98 of complexed ligand). The effect of the metal ion was also evaluated by variations of the NMR signals induced by the addition of Ni(II) ions. In the diagram of chemical shift variation on NH-Hβ correlations, induced by Ni(II) at pH 5.2 (Figure 6B), a large shift of His103 and relatively smaller shifts of aspartic (Asp110, Asp118) and glutamic (Glu117) acids are observed. Additionally, the comparison of the obtained ^1^H–^1^H TOCSY spectra evidenced strong shifts in the correlation signals of the NH-Hγ protons of Glu117, and weak perturbations on aromatic protons of histidines, which confirms the involvement of these residues in the binding (Figure S3A and S3C). Comparing the graph of the shifts at pH 5.2 of L1 (Figure 6A), and the graph of L2 (Figure 6B), larger shifts of aspartic acids and glutamic acid of ligand L2 are noticed. Most likely, this is due to a histidine mutation in position 102, leading to an increased proportion of acidic amino acid residues involved in nickel ion coordination. Ni(II) binding to this peptide causes chemical shift variations also in the case of Arg119 signals. This may be due to the electrostatic interactions between the positively charged amino acid residue of arginine and the negatively charged carboxyl groups deprotonated at this pH which may stabilize the complex. The loss of the next proton, in NiHL form, corresponds to the second histidine residue; the p*K*_a_ value of 6.31 is decreased in comparison to that of the free ligand (p*K*_a_ = 7.19). This implies the participation of this group in Ni(II) coordination. In the NMR spectra recorded at pH 7.4, the addition of Ni(II) ions causes a selective effect on protons of His106, His103, and His114 (Figures S1B and S3B). Moreover, the shift of NMR signals observed for the His aromatic protons strongly indicates the involvement of His in metal binding (Figure S3D). The next complex, NiL, with a p*K*_a_ value of 9.45 and maximum formation at pH 9.0, corresponds to the deprotonation of the imidazole nitrogen atom of the next histidine. A comparison of the p*K*_a_ value of this histidine in the complexed ligand to the same histidine residue of the free one (p*K*_a_ = 9.65) suggests that this residue does not participate in the binding. The UV–vis and CD spectra recorded at the pH range 2.4–7.2, do not show relevant changes, suggesting that the octahedral geometry is maintained (Figure S4A,B). The last 2 species (NiH_-2_L and NiH_-3_L) which dominate at pH 9–11, involve respectively two and three amide nitrogen in the metal coordination sphere. These results are in agreement with the spectroscopic data. In the UV–vis spectrum, the wavelength of maximum absorption blue shifts to 440 nm which confirms the changing geometry of the square planar geometry in the complex (Figure S4A).^40,41^ As the pH increases, imidazole nitrogen is gradually replaced by amide nitrogen thus in the last form NiH_-3_L, the binding mode is 4N {1N_im_, 3N_am–_}. In CD spectra, the band at 275 nm is characterized by N_am_^–^ → Ni(II) charge-transfer transition, and characteristic of square planar geometry, the negative Cotton effect at around 425 nm and positive Cotton effect at 500 nm are present at higher pH (Figure S4B).

### Ni(II) Complex of the Ac-_101_DHHLAAIVVDAIAHASEDRR_120_ (L3)

Ligand Ac-_101_DHHLAAIVVDAIAHASEDRR_120_ (L3) forms only equimolar complexes with Ni(II), which was confirmed by MS analysis (Figure S5). {[NiL]^3+^-H_2_O}^3+^ (*m*/*z* = 759.70) corresponds to the ion of the complex after the neutral loss of the water molecule. In the spectra we can also observe the presence of signals corresponding to free ligand L3 ([L]^3+^*m*/*z* = 747.02) and the complex adduct with the K^+^ ion ([NiL]^2+^+K^+^}^3+^*m*/*z* = 780.41).

The coordination of peptide to Ni(II) results in six different protonated species, starting from NiH_3_L at low pH. In this situation, five sites are deprotonated which belong to the C-terminal, acidic side chains of glutamic acid, and three aspartic acids (Table 3). In the diagram of chemical shift variation induced by Ni(II) at pH 5.2 (Figure 6C), a large shift of His103 and a relatively smaller shift of aspartic (Asp110, Asp118) and glutamic (Glu117) acids are observed. The suggested coordination sphere is also confirmed by the large shift variation of the correlation signals of NH-Hγ of Glu117 (Figure S6A) and the shift of the overlapped aromatic protons of histidines (Figure S6C). Ni(II) binding to this peptide causes chemical shift variations also in the case of Arg119 signals. The next deprotonations lead to the formation of NiHL. It is the most abundant complex at pH 7 (reaching 90% of the total nickel concentration). The following form, NiL, with maximum formation at pH around 8.2 corresponds to the deprotonation and coordination of the third histidine residue. This result is in agreement with a major difference between p*K*_a_ values of the histidine residue in the complex and in the free ligand (9.45 for the free ligand, 8.28 in the complex). In the NMR spectrum at pH 7.4 (Figures S6B and S1C), the addition of Ni(II) ions leads to the disappearance of the HN-Hβ correlations of the histidine signals which made it impossible to analyze the Ni(II)-coordination sphere at the tested pH accurately. Therefore, to confirm the participation of imidazole nitrogen in the coordination sphere, an aromatic region of the spectra was used (Figure S6D). The slight shifts in Hδ-Hε correlations of histidines strongly suggest the involvement of these side chains in Ni(II) binding. In the UV–vis and CD spectra recorded at pH 6.15–8.09, no significant changes occur (Figure S7A,B). The lack of d–d transition CD bands implies no main-chain coordination below pH 9, confirming the involvement of histidine residue donor atoms in the binding metal. The loss of the next three protons corresponds to the deprotonation of amide nitrogen atoms from the peptide backbone. Above pH 8, the NiH_-1_L form, with a p*K*_a_ value of 8.34, starts to appear. The maximum formation of this complex occurs at pH 9 when a strong CD band (λ_max_ = 280 nm) is observed for the N_am_^–^ → Ni(II) charge transfer transition. In the CD spectra recorded at pH 9–11, negative ellipticity around 420 nm and a positive one at 550 nm also appears, suggesting the formation of a square planar complex (Figure S7B).^40,41^ Two further deprotonations lead to the formation of NiH_-2_L and NiH_-3_L species which reach 50% and above 90% of the Ni(II) concentration at pH around 9.5 (p*K*_a_ = 8.93) and pH 11 (p*K*_a_ = 9.78), respectively. Their formation can be attributed to two further deprotonations of amides from the backbone. The lack of a difference in absorption between these pH values suggests no changes in the geometry of the complexes.

### Ni(II) Complex of the Ac-_116_DHHLAHIVLDAVAHAGEDAI_135_ (L4) peptide

Peptide Ac-_116_DHHLAHIVLDAVAHAGEDAI_135_ (L4) forms only equimolar complexes with Ni(II); in the MS, spectrum the following signals were detected: {[NiL]^3+^-H_2_O}^3+^, [L]^3+^, {[NiL]^3+^+H_2_O}^3+^, {[L]^3+^-H_2_O}^3+^ {[L]^2+^+Na^+^}^3+^, and {[NiL]^2+^+K^+^}^3+^ (Figure S8).

Potentiometric calculations suggest that in presence of Ni(II), L4 forms six complexes. The first observed species is NiH_4_L (Table 3 and Figure 4D) which is formed after deprotonation of the C-terminus and four residues of acidic amino acids. In this form, residues of Asp and Glu coordinate Ni(II) ions. This form starts at pH 4 and reaches the maximum at pH 5–6. NiH_3_L species formed as a result of the first histidine residue deprotonation. It starts at pH 5 and reaches the maximum at pH 6. Similar to NiH_4_L, it is not a significant form; the p*K*_a_ value of the first histidine residue decreases suggesting the involvement of this residue in metal binding. Analysis of the NMR spectra recorded at pH 5.2 (Figure S9A) and plot of chemical shift variations (Figure 6D) revealed that His121 may indeed strongly interact with the Ni(II) ion, which was confirmed by the chemical shift perturbations on aromatic protons of histidines (Figure S9C). In the charge transfer transition area of the UV–vis spectra recorded at pH 3.1–8.1, no relevant changes were observed, suggesting that the octahedral geometry is maintained (Figure S10A). As a result of the second histidine residue deprotonation, NiH_2_L species is formed. It starts at pH 5 and reaches the maximum at pH 6–7. Potentiometric calculations show that there is a significant decrease in the p*K*_a_ value of the histidine residue (6.8 in the free ligand and 6.06 in the complex) which suggests its involvement in Ni(II) binding. When the third histidine residue deprotonates, NiHL species is formed. According to the potentiometric calculations, NiHL is the most significant form at pH 7–8, and there is a big decrease in the p*K*_a_ value of the third histidine residue (7.3 in free ligand and 6.46 in complex, Table 3). This indicates that a third histidine residue binds Ni(II). Analysis of the both fingerprint and aromatic regions of the NMR spectra recorded at pH 7.4 (Figure S9B and S9D) and plot of chemical shift variations (Figure S1D) revealed that His121 and two other His residues are involved in Ni(II) binding. However, the NH-Hβ and Hδ-Hε correlations from His117, 118, 121 and 129 were overlapped. The UV–vis and CD spectra recorded at pH range 3.1–8.1, do not show relevant changes, thus suggesting that the octahedral geometry is maintained (Figure S10A,B). However, it is possible that two or even three imidazole nitrogen are now bound to nickel. When the last histidine residue along with one of the amide nitrogen deprotonate, another complex form is created: NiH_-1_L. That form dominates at pH 9 and exists mainly in the 9–10 pH range. Analysis of the CD spectra recorded at pH 9–12 revealed that a huge band increase in charge transfer transition area occurs (λ_max_ = 275 nm). It means that N_am_^–^ → Ni(II) transition took place and, with the increasing pH, three amide nitrogen atoms have been involved in Ni(II) binding, replacing three His residues in the coordination sphere of Ni(II) in the end.^40,41^ The spectroscopic parameters of NiH_-1_L are consistent with a 4N {1N_am_^–^, 3N_im_} donor set. There is a weak band in the d–d transition area of the UV–vis spectra with a maximum at 430 nm and d–d bands at 425 and 550 nm (negative and positive Cotton effect) in the CD spectra (Figure S10B), which confirm that the formation of square planar geometry of the NiH_-1_L species starts. The last occurring complex species forms when two other amide nitrogen atoms deprotonate and bind Ni(II): NiH_-3_L of square planar geometry. It starts at pH 9 and reaches the maximum at pH 11. The presence of this species was confirmed by UV–vis and CD spectra analysis. The results indicate that indeed the three amide nitrogens may be involved in nickel ion binding. Finally, the spectroscopic parameters are consistent with a 4N {3N_am_^–^, 1N_im_} donor set.

At the DFT level of theory, we have found multiple coordinated Ni(II) complexes for L1 and L4 model peptides. Investigated ligands form 3N type of complexes, where three Ni(II)···imidazole rings of His102, His106, and His114 residues are involved as well as two Ni(II)···O supporting the interaction. The Ni(II)···Ac-_101_DHHLAHIVVDAIAHASEDRR_120_ (L1) complex forms a binding pocket with well-defined metal—ligand distances with an average bond length of 1.95 Å (Table 4).

**Table 4:** Metal—Ligand Distances in Angstroms

	Ni(II)···Ac-_101_DHHLAHIVVDAIAHASEDRR_120_ (L1)	Ni(II)···Ac-_116_DHHLAHIVLDAVAHAGEDAI_135_ (L4)
Ni(II)···.N (H2)	1.940	1.982
Ni(II)···N (H6)	1.975	1.984
Ni(II)···N (H14)	1.953	1.952
Ni(II)···O (E17)	1.942	1.971
Ni(II)···O (D18)	2.231	2.496

The binding pattern of the Ni(II)···L4 complex is identical – three imidazole rings from H102, H106, and H114 interact with the cation. Please note that the average Ni(II)···N_im_ bond length in Ni(II)···L4 (1.97 Å) is longer than in Ni(II)···L1 (1.95 Å) complex as well as both “supporting” interactions with oxygen atoms. Due to different residue sets in investigated ligands, the direct comparison of energy of the complexes is not possible. Although, the metal—ligand bond length may be a good indicator of interaction energy as long as we take into account the same type of interactions. One could expect that Ni(II)···L1 shall be more stable than Ni(II)···L4 due to stronger metal–ligand interactions displayed by shorter metal–ligand distances.

### Intramolecular Hydrogen Bonds

The intramolecular hydrogen bonds (HBs) can provide additional stability to the complexes with peptide ligands. The most common HBs we observed in such complexes were O···H–N. The proton acceptor pair can be provided by the ligand backbone as well as the side chains. The proton acceptor is commonly an oxygen atom from the carbonyl group. The intramolecular O···H–N HBs of the backbone bring ∼5 kcal/mol per HB to stability; moreover, it can be expected that such interaction can provide and/or stabilize helical secondary fragments of the ligand. As expected, we have found helical fragments in both complexes. In the Ni(II)···L1 complex, we have found an α-helical fragment with a short 3–10 helix beginning as shown in Table 5. The hydrogen bonds form short cooperative chains, and the pass between 3 and 10 and α helixes is generated via bifurcated hydrogen bonds this is typical for mixed 3–10 and α helixes.

**Table 5:** Hydrogen Bonds of the Ni(II)···Ac-_101_DHHLAHIVVDAIAHASEDRR_120_complex (L1)

residue	H···PA [Å]	PD-H···PA [deg]	fragment	
D1···H3	1.819	157.9	O···H–N	
D1···L4	2.800	152.1	O···H–N	
D1···H2	1.762	170.3	O (side chain)···H–N	
H2···A5	1.923	153.8	O···H–N (α helix)	
L4···H6	1.982	164.2	O···H–N (3–10 helix)	
H6···V9	2.028	160.6	O···H–N (α helix)	
V8···A11	1.770	166.0	O···H–N (α helix)	
V9···I12	2.357	169.7	O···H–N (α helix)	
D10···A13	1.861	151.0	O···H–N	
D10···H14	2.087	157.4	O···H–N	
A11···A15	1.700	158.1	O···H–N	
I12···R20	1.891	157.8	O···H–N (side chain)	
A13···S16	1.682	161.2	O···H–N (side chain)	
H14···E17	1.884	163.8	O···H–N	
H14···E17	1.627	1774	H–N (side chain) ··· O (side chain)	
A15···D18	1.746	166.4	O···H–N	
S16···R19	1.791	165.4	O···H–N	
R19···E17	2.016	135.6	N–H (side chain) ··· O (bifurcated)	
R19···E17	1.916	130.2	N–H (side chain) ··· O (bifurcated)	
R20···Ac	1.774	170.5	N–H (side chain)···O	
R20···D18	1.730	160.8	N–H (side chain)···O (side chain)	

The Ni(II)···L4 complex also builds a rich intramolecular H-bonds network (Table 6); however, the number of HBs is about half of the number that we have found in the Ni(II)-L1 complex. As a result of fewer intramolecular hydrogen bonds, fewer regular, helical structure fragments are present in the Ni(II)···L4 complex.

**Table 6:** Hydrogen Bonds of the Ni(II)···Ac-_116_DHHLAHIVLDAVAHAGEDAI_135_ (L4) Complex

residue	H···PA [Å]	PD-H···PA [°]	fragment	
D1···H3	1.971	136.3	O···H–N (3–10 helix)	
H2···L4	1.858	169.0	O···H–N (3–10 helix, bifurcated)	
H2···A5	2.001	152.8	O···H–N (α helix, bifurcated)	
A15···D18	1.844	157.0	O···H–N	
D18···A15	1.611	172.3	O_1_ (side chain)···H–N	
D18···H14	1.651	174.6	O_2_ (side chain)···H–N	
A15···E17	2.032	162.4	O···H–N (3–10 helix)	
GS16···I20	1.855	155.3	O (side chain)···H–N(side chain)	
S16···I19	1.873	177.5	O···H–N (α helix, bifurcated)	
S16···R19	1.955	154.2	O···H–N (side chain, bifurcated)	

Both ligands have similar fragments of the sequence; however, in the terms of stability caused by intramolecular hydrogen bonds, the presence of two arginines is crucial for the stability of the Ni(II)···L1 complex. Due to the extended side chains and arginines, N–H proton-containing donors are excellent partners to create hydrogen bonds. Interestingly, in the Ni(II)···L1 25% of hydrogen bonds are built with arginines that are only 10% of the analyzed peptide sequence. This study revealed that Ac-_101_DHHLAHIVVDAIAHASEDRR_120_ (L1) and Ac-_116_DHHLAHIVLDAVAHAGEDAI_135_ (L4) ligands form thermodynamically stable complexes with the Ni(II) cation.

Both complexes create 3N type interactions with His102/117, 106/121, and 114/129 residues supported by two Ni(II)···oxygen interactions. The Ac-_101_DHHLAHIVVDAIAHASEDRR_120_ ligand forms a more stable complex with Ni(II) in comparison to the Ac-_116_DHHLAHIVLDAVAHAGEDAI_135_ ligand. Both complexes have a very rich network of hydrogen bonds. The complex of Ac-_101_DHHLAHIVVDAIAHASEDRR_120_ doubles the number of hydrogen bonds compared to Ni(II)···Ac-_116_DHHLAHIVLDAVAHAGEDAI_135_, mainly due to interactions provided by the side chains of Arg residues.

### Study on the Secondary Structure of Complexes

The CD spectrum of free L1 shows that according to the literature, the secondary structure of the peptide is probably a mixture of α-helix and other forms: random coil/β-sheet (Figure S11).^43^ The increase of pH enhances the participation of the beta-sheet/random coil form. The addition of Ni(II) ions does not significantly impact the secondary structure, and the trend of α-helix formation with the decreasing pH is maintained (Figure S12). Analogical results were obtained for L4. CD results for L2 and L3 revealed that these two mutants did not form secondary structures.

## Discussion

In this work, we examined the pH-dependent properties of Ni(II)-L systems of SmtB, BigR4, and mutants of BigR4 α5 domains. The stoichiometry, stability, and geometry of complexes were investigated by a variety of analytical methods. Studies on the impact of point mutations and NMR analysis allowed us to determine the overall Ni(II)-binding motif of the α5 domain of SmtB and BigR4.

**Figure 8:**
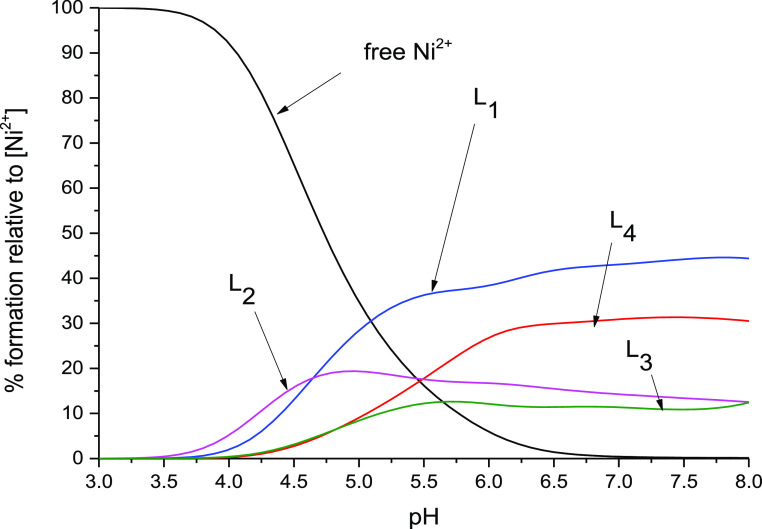
Competition plots between L1, L2, L3, and L4 peptide complexes with Ni(II) ions, expressing complex formation in a hypothetical solution where two or three peptides and one metal ion are mixed together (M/L1:L2:L3:L4 = 1:1:1:1:1). The calculation is based on the potentiometric data present for the studied systems in Table 2.

**Figure 9:**
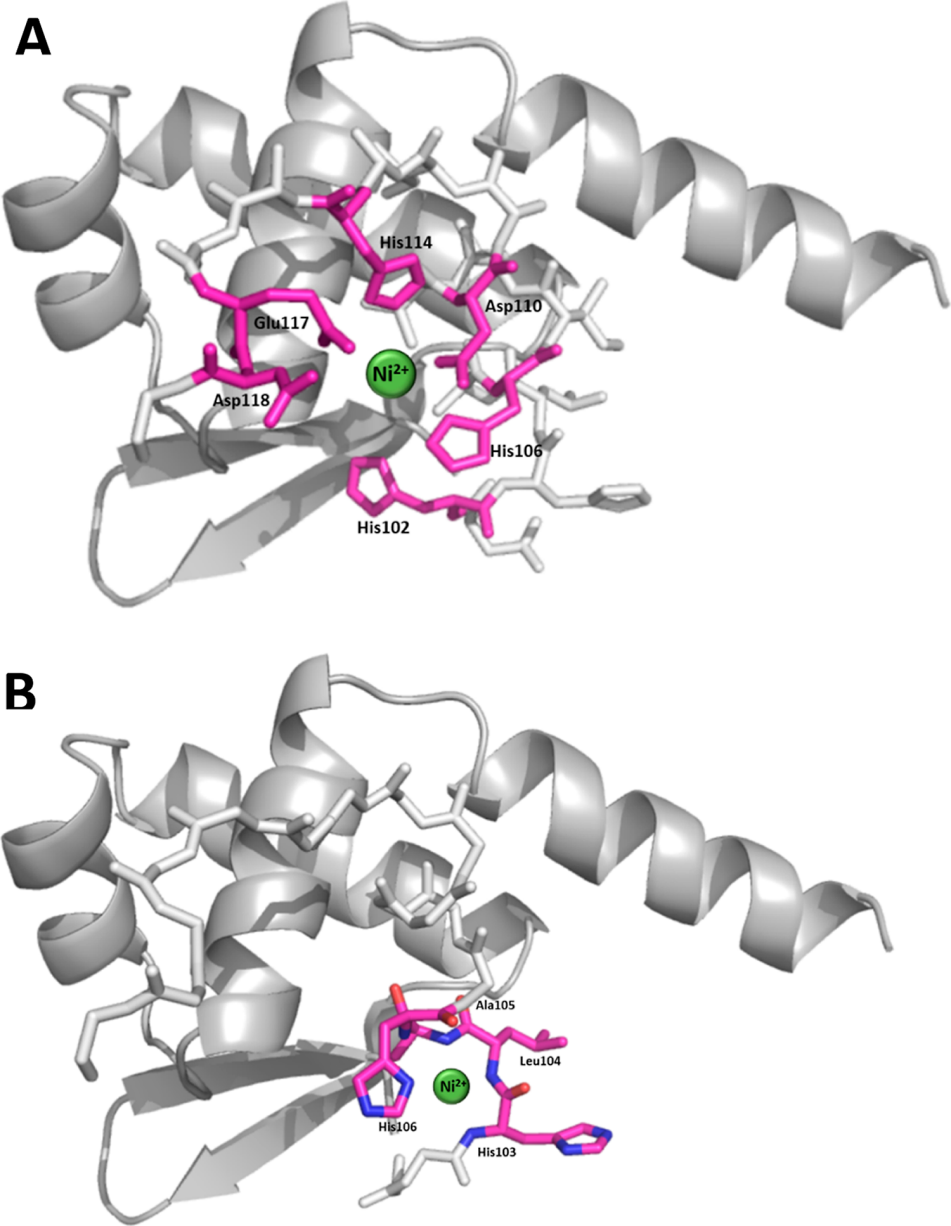
Proposed coordination sphere for the nickel(II)-L1 complex at (A) pH 7.4 and (B) pH 10. The structure of the BigR4 protein is based on simulation by Phyre2. Figures were generated using PyMOL.

The data obtained by potentiometric titrations revealed that a maximum of 3 His residues are involved in Ni(II) binding of L1-L4. The coordination sphere of the metal ion is completed by residues of acidic amino acids. In the case of L2 and L3, a maximum of 2 His residues bind Ni(II). On the basis of stability constants of complexes, we were able to draw the competition plot (Figure 8), which describes complex formation at different pH values in a hypothetical situation when the mixture of equimolar ligands L1-L4 compete for Ni(II) ion in solution (M/L1:L2:L3:L4 = 1:1:1:1:1). The competition plot (Figure 8) revealed that L1 (α5 domain of BigR4) forms more stable complexes with Ni(II) rather than with L2, L3 (mutants), or L4 (α5 domain of SmtB) in a wide pH range. These results strongly suggest that His102 and His106 in the sequence of BigR4 are involved in metal binding. To support the abovementioned, we observed a decrease in the stability of Ni(II)-L in the absence of His102 and His106. This was also confirmed by NMR analysis. The chemical shift variations observed on His aromatic protons at pH 5.2 (Figures 5C, S3C, S6C, and S9C) are smaller for L4 and L3 complexes, and more relevant for L2 and then L1 complexes which correlate well with the competition plot (Figure 8). Additionally, at pH 5.2 a significant shift of signals corresponding to NH-Hβ correlations of Asp110, Glu117, and Asp118 of L1 are observed in the presence of Ni(II) ions, suggesting the involvement of these amino acid residues in Ni(II) binding (Figure 5A). Additionally, an enormous shift in NH-Hβ correlations of His106 and smaller ones in His114 and His102/103 (overlapped signals) was observed, suggesting that in this pH, only one histidine residue binds Ni(II) – His106. This correlates well with potentiometric data. It also suggests that His114 and His102 or 103 are close to Ni(II) and may bind metal ions at higher pH. At pH 7.4 (Figure 5B), NH-Hβ correlations of acidic amino acid residues partially vanished in comparison to signals of His106 and His102, 103, and 114 (overlapped signals). This leads to a conclusion that with increasing pH, His residues are more involved in Ni(II) binding. The study on mutants helped establish that His102, rather than His103, binds metal ions, and that both His102/106 strongly interact with Ni(II). This was confirmed by analysis of NMR plots (Figures 6A, S1A) and NMR spectra. The chemical shift variations observed on His aromatic protons at pH 7.4 (Figures 5D, S3D, S6D, and S9D) are much more significant for L1 and L4 (original fragments of proteins) than for L2 and L3 (mutants) – Ni(II)-induced shifts increase in the following order: L3, L2, L4, and L1. This result indicates distinctly that the deletion of His102 and 106 decreases the interaction of peptides with Ni(II), and that His106 is more important for Ni(II)-complex stability. On the other hand, it shows that L1 forms a more stable Ni(II)-complex than L4 which correlates with the competition plot (Figure 8). It is commonly observed that when an amino acid binds a metal ion, the signals corresponding to correlations of protons of this amino acid, but also its neighbors, are shifted or broadened.^44,45^ In this particular case, a significant shift of signals at pH 5.2 of Ile107 and Ala111 in presence of Ni(II) indicates that His106 and Asp110 bind Ni(II) (Figures 6A and S1A). In order to establish which of these two histidines, His102 or His103, also binds Ni(II), we have carefully investigated the shifts of correlations of Asp101 and Leu104. Both signals were shifted in the presence of Ni(II) at pH 5.2, and this shift decreased at pH 7.4. Interestingly, in the plot for L4 (Figure 6D), we observed a similar situation while in the plots for mutants—L3/L4 (Figure 6B,C)—the shift of signals of Leu104 vanished at pH 5.2 and 7.4. It is worth mentioning that shifts of signals of Asp101 decreased in the case of L2 and vanished in the case of L3 at pH 5.2. The opposite situation was observed at pH 5. The shift of Leu104 correlations is observed in the spectra of L1 and L4 because of the close proximity of His102 involved in Ni(II) binding. His102 is replaced with the Ala residue in L2, and it is the only situation when His103 is involved in Ni(II) binding. This analysis allowed us to conclude that the most probable Ni(II)-binding sites of L1 are: His102, 106, and 114; Glu117; and Asp110 and 118 (Figure 9).

In the case of L4, we observed a similar situation in the NMR spectra: in the presence of Ni(II) ions at pH 5.2, correlations of acidic amino acid residues and His102/103 (signals overlapped) were shifted. However, a correlation signal of His106 and His114 has vanished. It suggests that one of these two amino acids is more affected by the presence of Ni(II) ions. Most probably—His106 binds Ni(II) by analogy to L1. The shift of neighboring Ile residue signals strongly supports this conclusion. At pH 7.4, the signals corresponding to His102, 103, and 106 are overlapped and shifted, while the biggest shift was observed for correlation signals corresponding to His106. The shift of signals corresponding to Asp110, 118, and Glu117 was decreased. It may be concluded that in both, L1 and L4, the same amino acid residues are involved in Ni(II) binding and the binding motif is as follows: HX_3_HX_3_DX_3_HXXED.

Our studies also provide some interesting data concerning the comparison of the full-length ArsR-SmtB type protein and model peptides of the α5 domain structure. The scheme of the α5 domain in ArsR-SmtB family representatives (Figure 2) was created on the basis of reports concerning structures of whole proteins studied with the use of XRD or a structure of a folded protein in a solution studied with the use of NMR.^46,47^ To obtain a protein crystal or a properly folded protein in solution, particular conditions must be reached. Usually, a variety of conditions of crystallization must be tested before, for example, salt concentration, type of buffer, the addition of polymers, and so forth, and even then some proteins do not fold properly. In our study of Ni(II)-L complexes secondary structure (Figures S11 and S12), we did not use a buffer solution suitable for proteins—the type of solvent and salt concentration were adjusted regarding the potentiometric titrations experiments or NMR. Our studies were focused not on proteins but on short fragments of proteins (approximately 20 AA). The addition of, for example, PEG usually stabilizes the folded structure of a protein or its oligomeric state; however, we did not use any stabilizing agents in order to maintain similar sample preparation for all used techniques. Our results show the difference between the secondary structure of the Ni(II)-complex of the isolated a5 domain, and the analogical complex in the whole protein (Figures S11 and S12).

## Conclusions

In this study, we have shown that L1 and L4 possess the same metal-binding motif, and their complexes with Ni(II) are characterized by the same equimolar stoichiometry and geometry—octahedral (pH < 9) or square planar (pH ≥ 9). The difference between these two ligands is in the stability of complexes. L1 forms more stable complexes with Ni(II) in a wider pH range than L4 despite the same number and character of donor atoms. To explain the difference in stability of complexes, we must keep in mind that both ligands have different primary structures. Particular amino acid residues may provide additional stability to complexes despite them not being directly involved in metal ion binding, for example, by forming a net of electrostatic interactions with other amino acid residues. DFT calculations revealed that in the case of L1, two Arg residues at the C-terminus indeed provide a net of hydrogen bonds that stabilize the whole structure of the complex. In the case of L4, there are no Args or other residues which could additionally affect the stability of its complexes with Ni(II).

The data obtained in this research shows intriguing features of the coordination chemistry of the α5 domain of SmtB (*M. tuberculosis*) and BigR4 (*M. smegmatis)*that strongly encourage further investigation of interactions between metal ions and particular histidine-rich domains of proteins engaged in virulence. We have shown that the α5 domain of SmtB and BigR4 has different binding motifs for Ni(II) and Zn(II) (Table 1). An explanation of this phenomenon may be that the nickel coordination number in complexes is 6 while for zinc it is usually 4. It makes the geometry of Zn(II) complexes with coordination number = 4 tetrahedral, while in the case of Ni(II) with coordination number = 6—it is octahedral—which forces the proper arrangement of peptide and its amino acid residues to fit the coordination sphere of the metal ion. Moreover, we have demonstrated that despite the same metal-binding motif, ligands with slight variations in the primary structure may form relatively stable complexes. The presence of Arg and similar residues providing additional electrostatic interactions may strongly stabilize the structure of the complex such as zinc fingers, as observed before.^48^ However, in this research, for the first time, we have established that this also applies to two very similar α5 domains of SmtB and BigR4. Why do these two proteins, with the same biological role as transcription activators in the presence of metal ions, have different affinity to Ni(II) ions and probably for Zn(II) ions? Is it correlated with *M. tuberculosis* pathogenicity and nonpathogenicity of *M. smegmatis*? To answer these questions, further research into the bioinorganic chemistry of SmtB/BigR4 must be done. Our concluded research provides some interesting leads for understanding the process of metal homeostasis in *Mycobacteria* and may be an interesting input for progressing antiTB drug development based on metal ions as an alternative to traditional antibiotics.

## Supporting Information Available

Plot of Ni(II)-induced chemical shift variation; mass spectra; NMR spectra; and UV–vis and CD spectra (PDF)
