# Supplementary material for: Coordination Properties of the Zinc Domains of BigR4 and SmtB Proteins in Nickel Systems—Designation of Key Donors
Source: Inorg Chem. 2022 Jun 13;61(25):9454–68. doi: 10.1021/acs.inorgchem.2c00319 (PMC9241078; doi:10.1021/acs.inorgchem.2c00319)
Supplement: Supplementary file 1 — ic2c00319_si_001.pdf [file ic2c00319_si_001.pdf]

Supporting Information

Coordination properties of the zinc domains of BigR4 and SmtB proteins in nickel systems - designation of key donors

Anna Rola,[1] Paulina Potok,[1] Robert Wieczorek,[1] Magdalena Mos,[2] Elżbieta Gumienna-Kontecka,[1] Sławomir Potocki[1]

slawomir.potocki@chem.uni.wroc.pl

[1] Faculty of Chemistry, University of Wroclaw, 14 Joliot-Curie St., 50-383 Wroclaw, Poland

[2] WMG, International Manufacturing Centre, University of Warwick, Coventry, CV4 7AL, United Kingdom

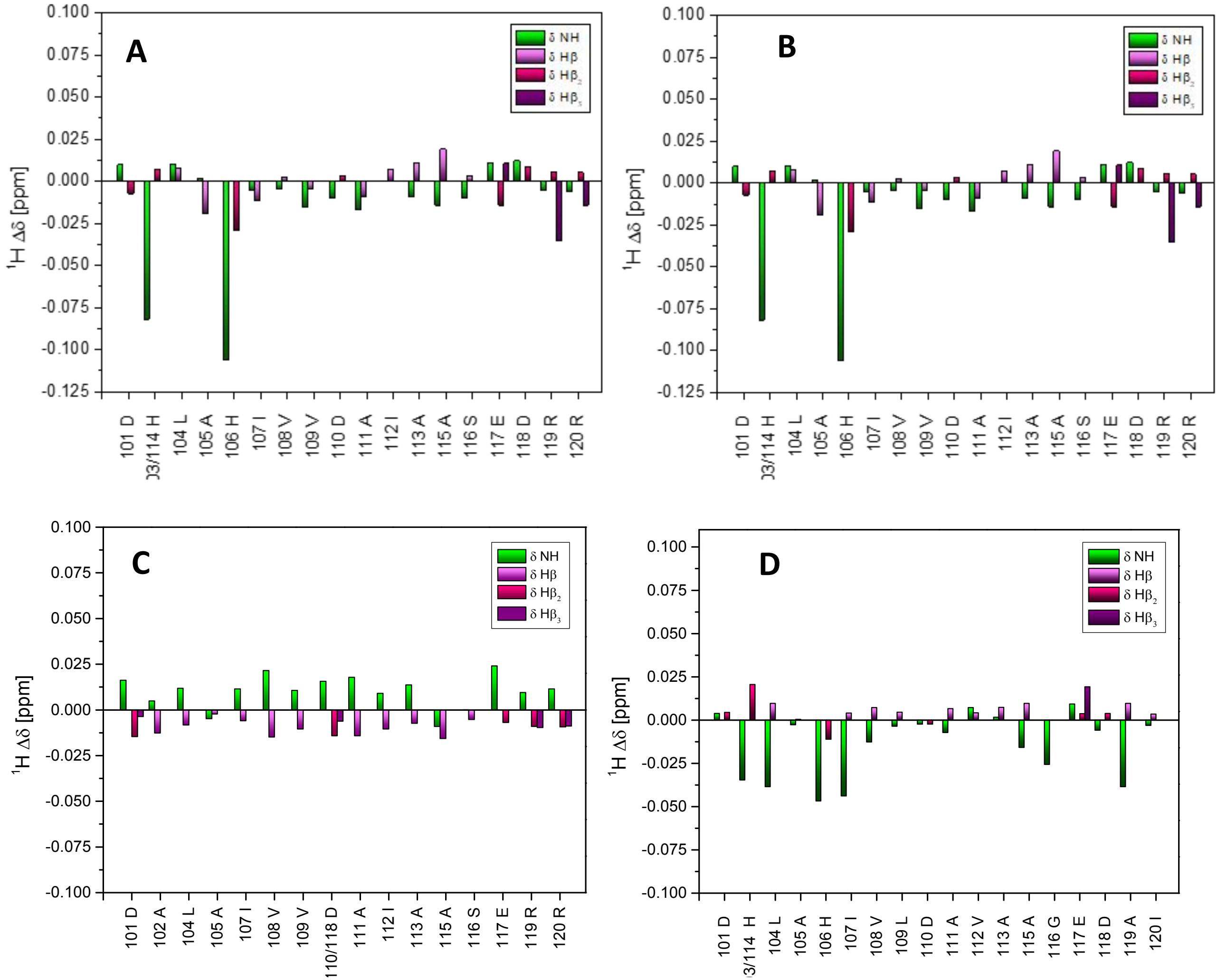

Fig. S1. Ni(II)-induced chemical shift variation at pH 7.4 of the selected proton of (A) L1, (B) L2, (C) L3, (D) L4.

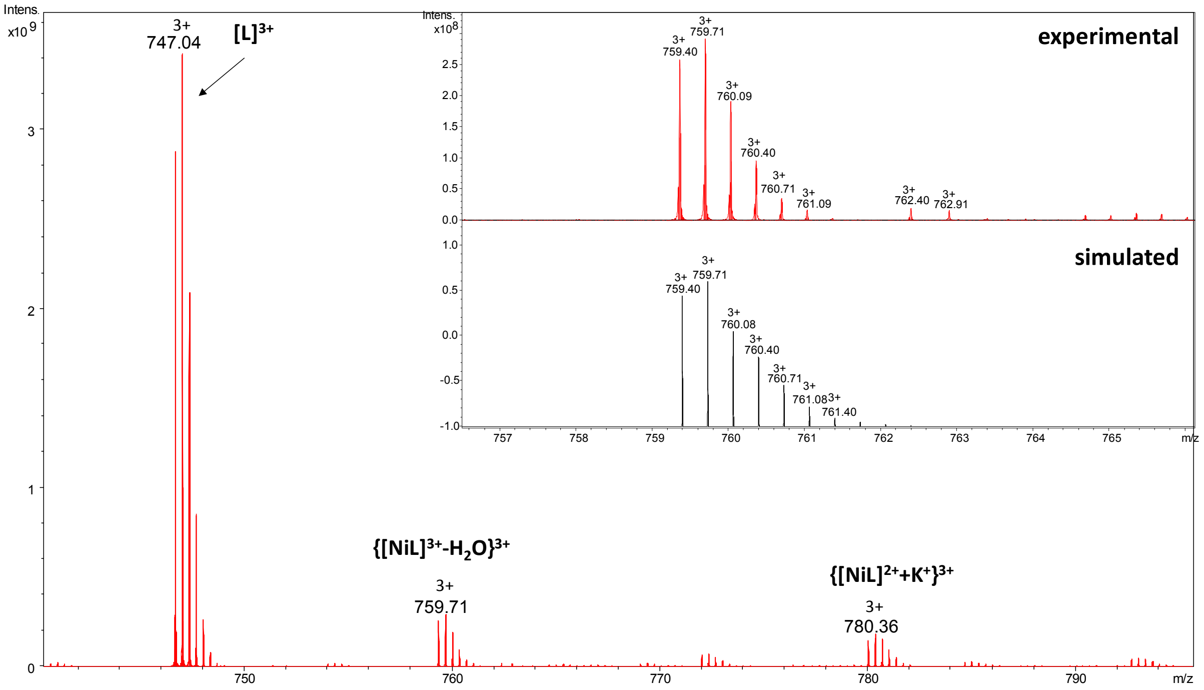

Fig. S2. ESI-MS spectrum of metal-ligand system composed of Ac-$_{101}$DAHLAHIVVDAIAHASEDRR$_{120}$ (L) and nickel(II) ions in the m/z 720-840 range at pH 7.4 [M:L = 1:1]. The simulated and experimental isotopic distribution spectra of peak at m/z= 759.71 are presented in the right corner

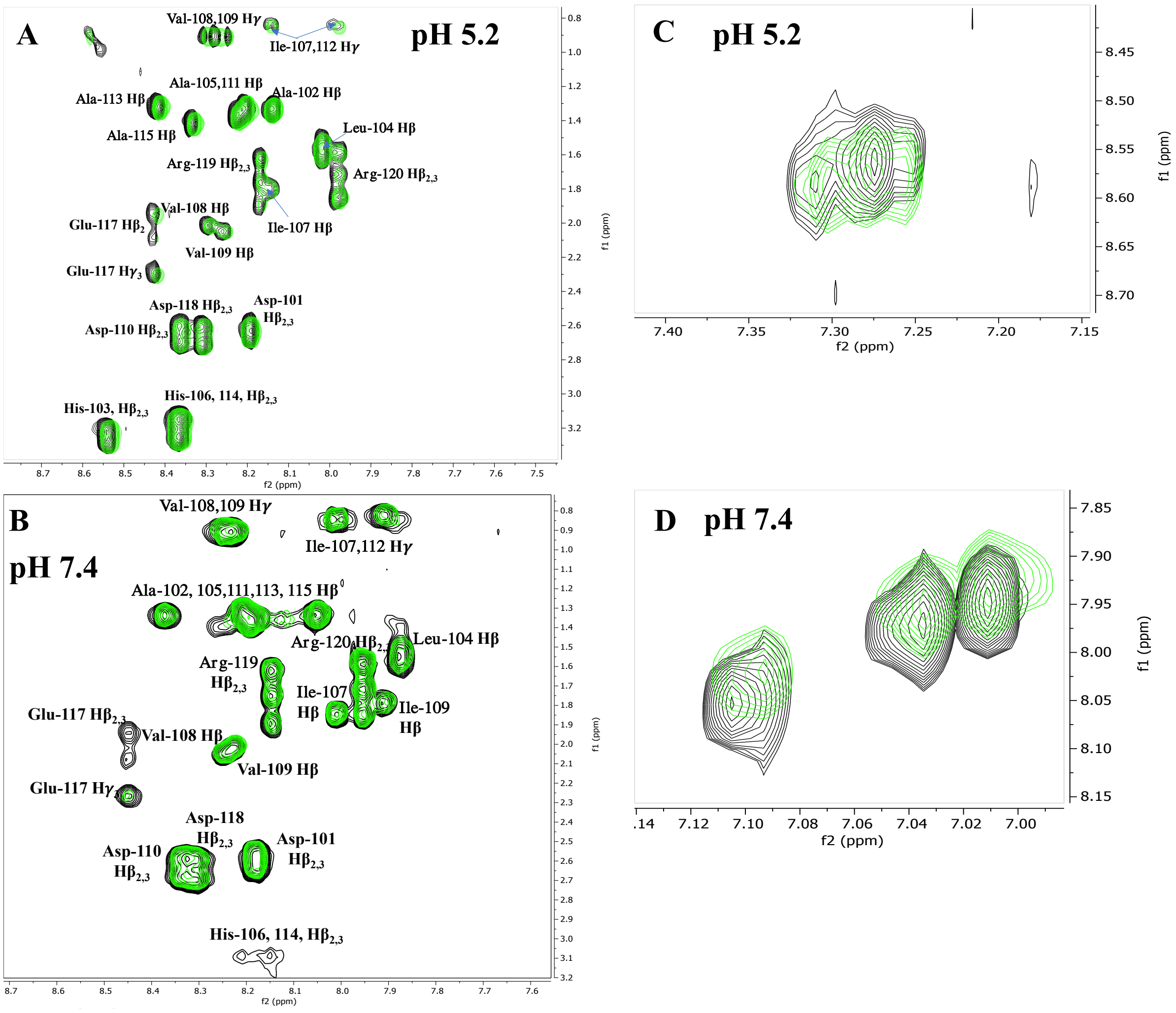

Fig. S3. ^{1}H–^{1}H **TOCSY NMR spectra of a fragment of the ligand (black) and the Ni(II) complex (green) with the ligand Ac-$_{101}$DAHLAHIVVDAIAHASEDRR$_{120}$ at pH=5.2 (A), (C), and at pH=7.4 (B), (D); finger print region – left, aromatic region – right; M:L=0.4:1, T = 298 K.**

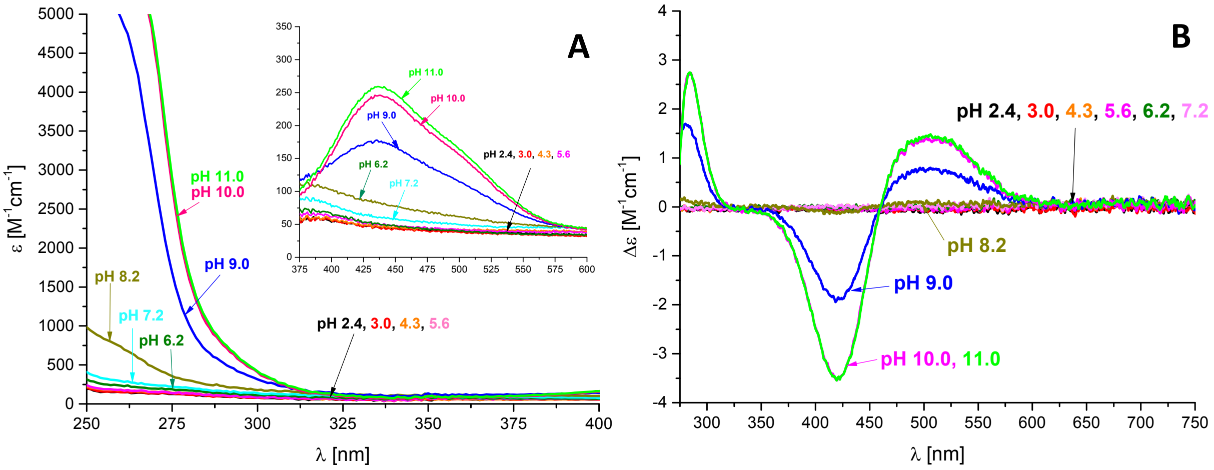

Fig. S4. UV-VIS (A) and CD (B) spectra of Ni(II) complexes with Ac-$_{101}$DAHLAHIVVDAIAASEDRR$_{120}$ (L2) peptide over the pH range 2-11. Conditions: T = 298 K, metal to ligand ratio = 1:1; [Ni(II)] = 2.5 x 10^{-4} M.

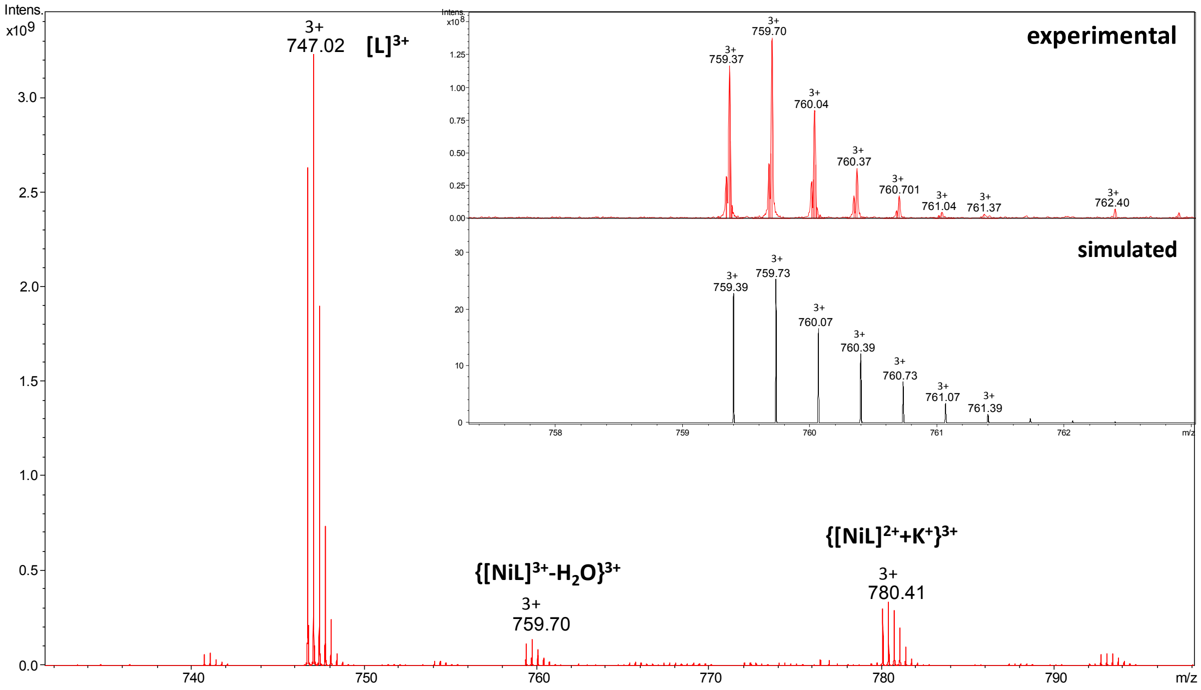

Fig. S5. ESI-MS spectrum of metal-ligand system composed of Ac-$_{101}$DHHLAAIVVDAIAHASEDRR$_{120}$ (L) and nickel(II) ions in the m/z 730-800 range at pH 7.4 [M:L = 1:1]. The simulated and experimental isotopic distribution spectra of peak at m/z= 759.70 are presented in the right corner

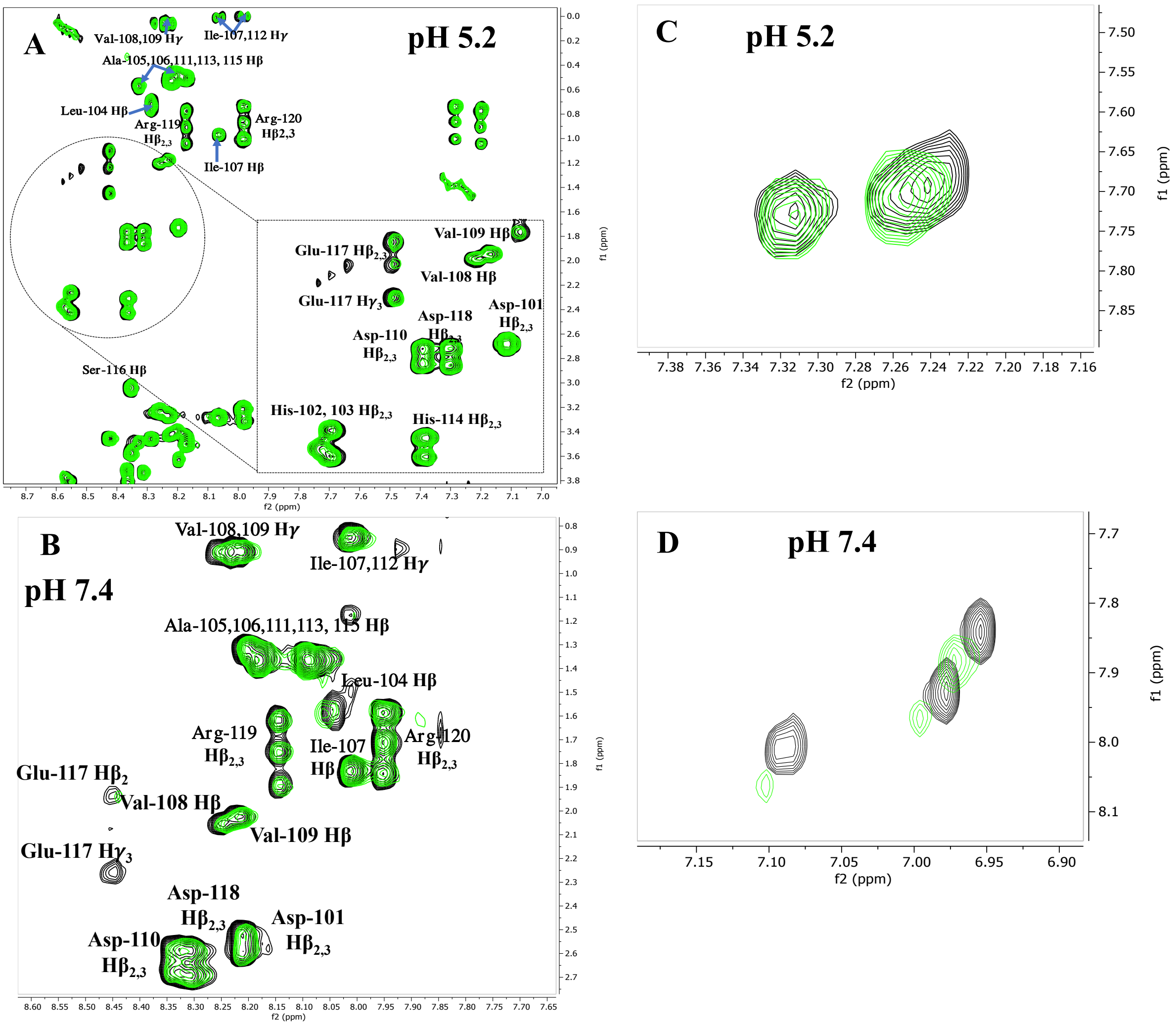

Fig. S6. ^{1}H–^{1}H TOCSY NMR spectra of a fragment of the ligand (black) and the Ni(II) complex (green) with the ligand Ac-$_{101}$DHHLAAIVVDAIAHASEDRR$_{120}$ at pH=5.2 (A), (C) and at pH=7.4 (B), (D); finger print region – left, aromatic region – right; M:L=0.4:1, T = 298 K.

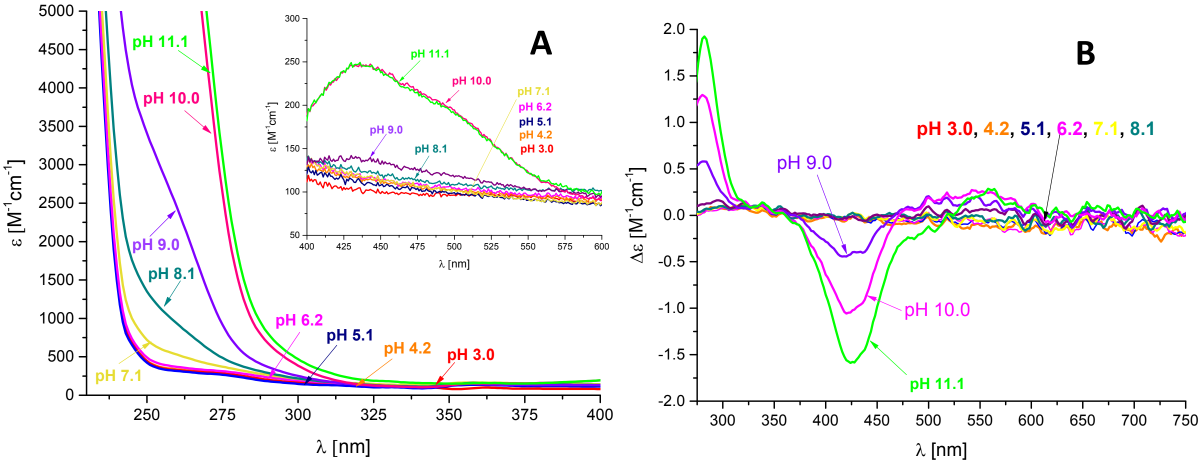

Fig. S7. UV-VIS (A) and CD (B) spectra of Ni(II) complexes with Ac-$_{101}$DHHLAAIVVDAIAHASEDRR$_{120}$ (L3) peptide over the pH range 2-11. Conditions: T = 298 K, metal to ligand ratio = 1:1; [Ni(II)] = 2.5 x 10^{-4} M.

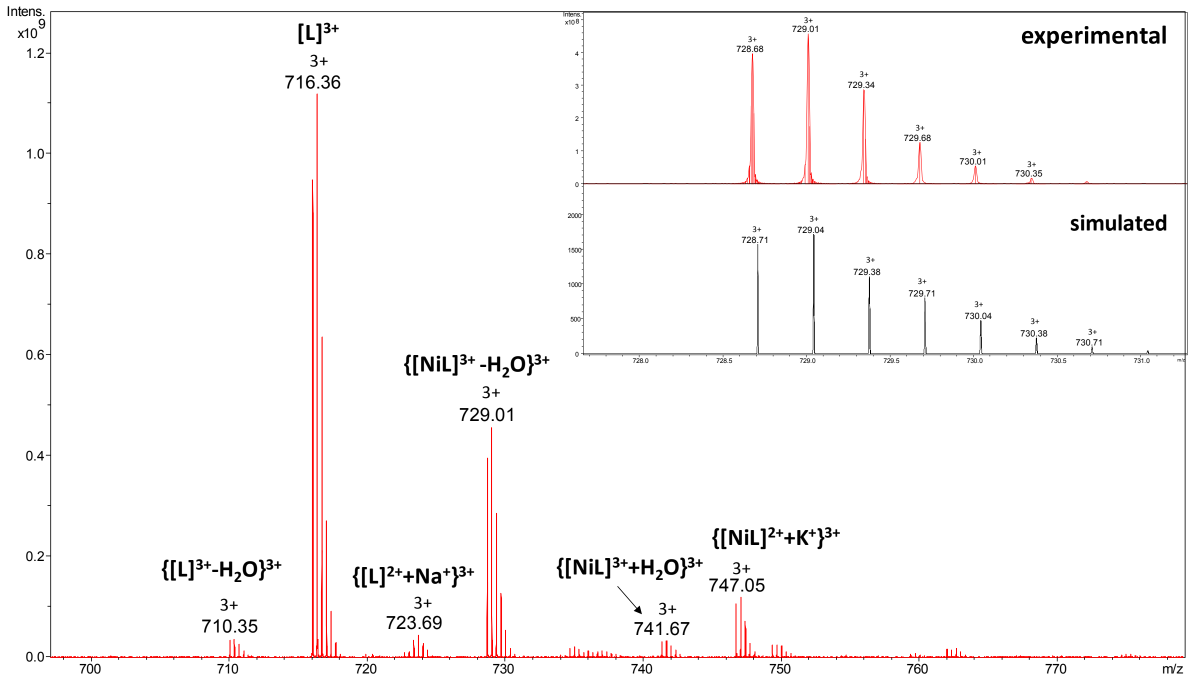

Fig. S8. ESI-MS spectrum of metal-ligand system composed of Ac-$_{116}$DHHLAHIVLDAVAHAGEDAI$_{135}$ (L) and nickel(II) ions in the m/z 700-780 range at pH 7.4 [M:L = 1:1]. The simulated and experimental isotopic distribution spectra of peak at m/z= 729.01 are presented in the right corner.

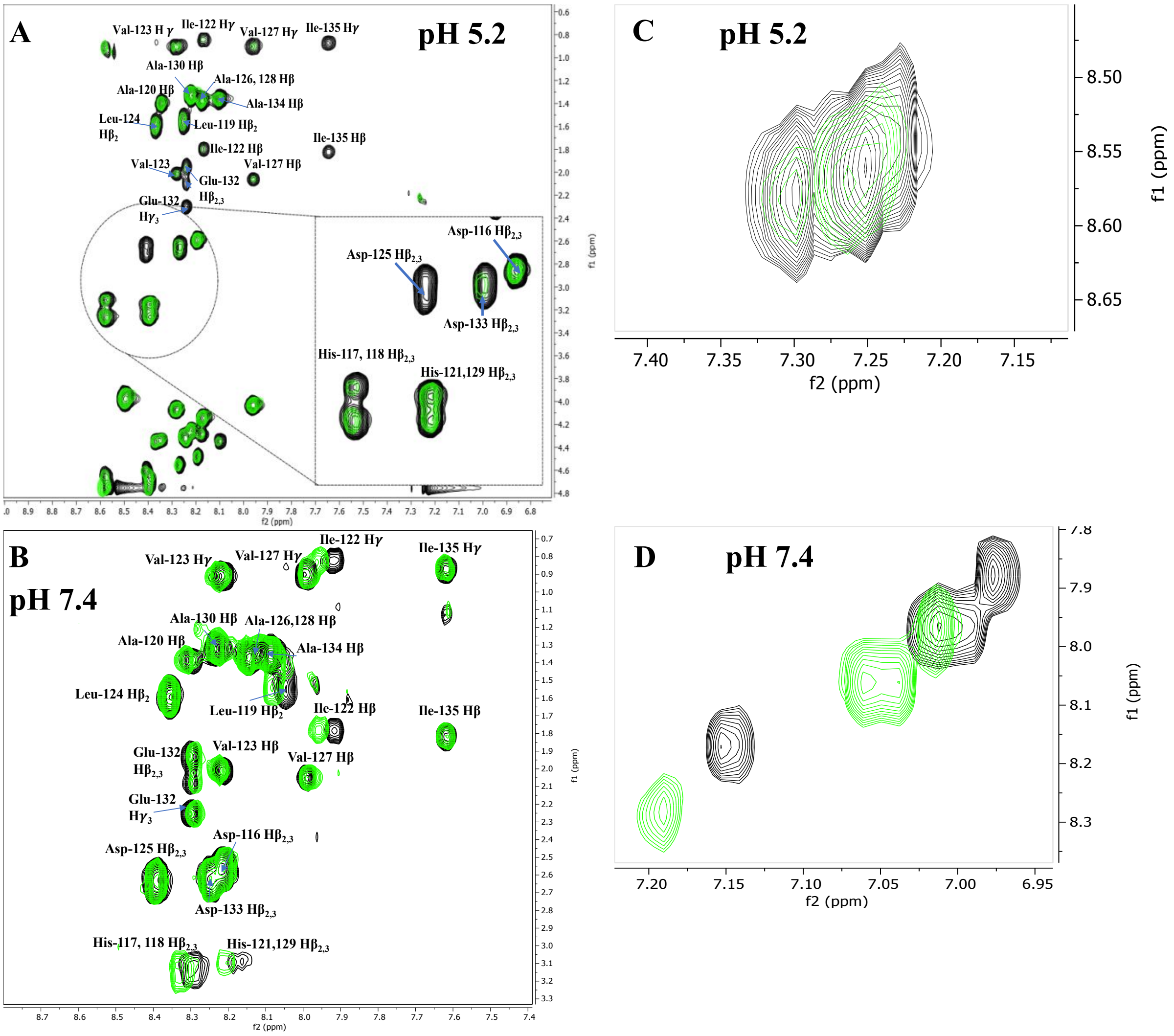

Fig. S9. ^{1}H–^{1}H TOCSY NMR spectra of a fragment of the ligand (black) and the Ni(II) complex (green) with the ligand $_{116}$DHHLAHIVLDAVAHAGEDAI$_{135}$ at pH=5.2 (A), (C) and at pH=7.4 (B), (D); finger print region – left, aromatic region – right; M:L=0.4:1, T = 298 K.

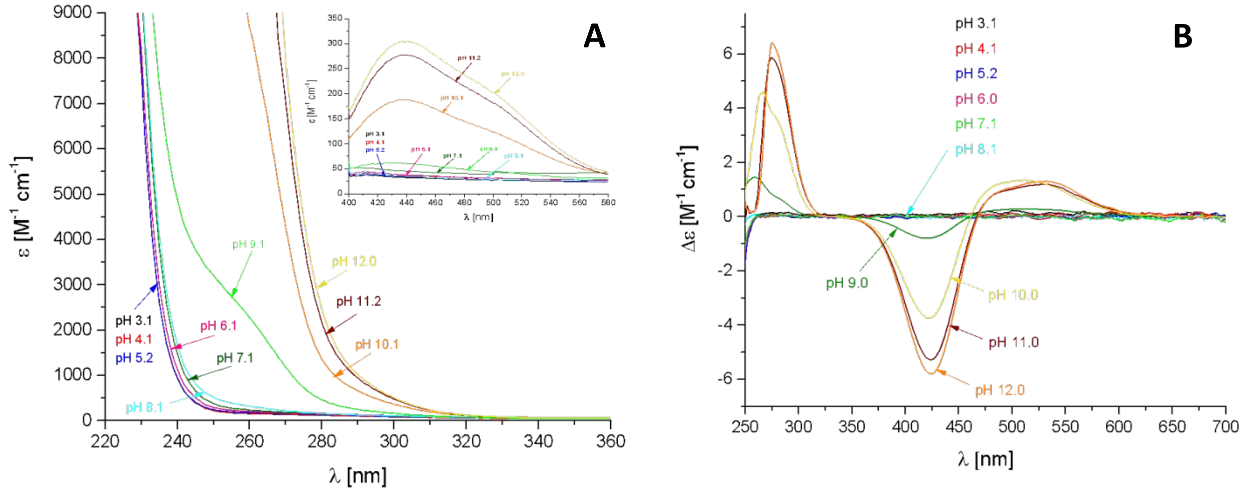

Fig. S10. UV-VIS (A) and CD (B) spectra for Ni(II) complexes of the Ac-$_{116}$DHHLAHIVLDAVAHAGEDAI$_{135}$ (L4) peptide. Metal to ligand ratio = 1:1; [Ni(II)] = 4.5 x 10^{-4}

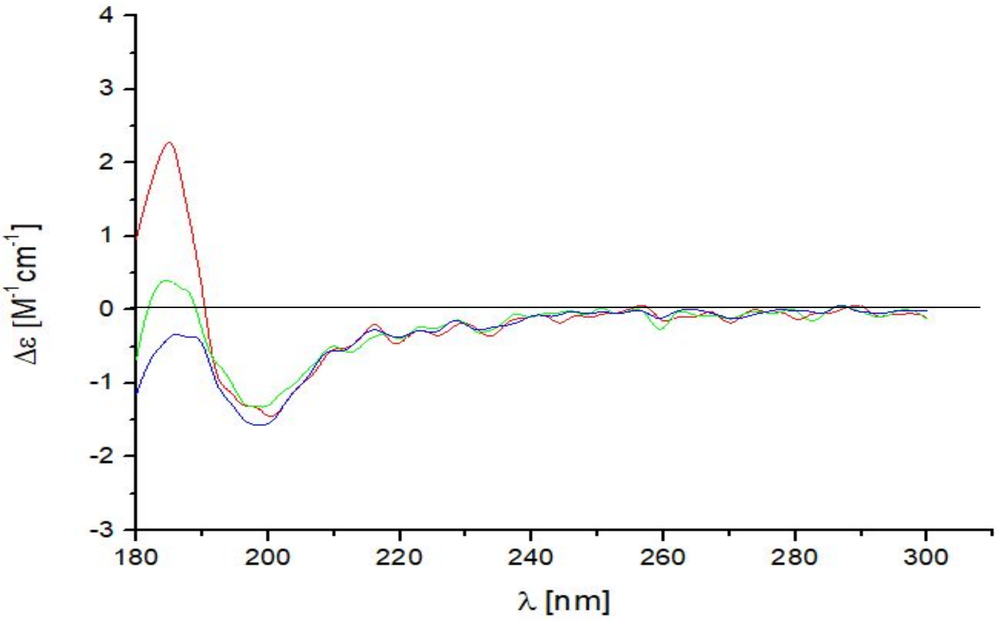

Fig. S11. CD spectrum at nm 180-320 range of free Ac-$_{101}$DHHLAHIVVDAIAHASEDRR$_{120}$ peptide (L1) at pH: 5 (red), 7 (green), 10 (blue).

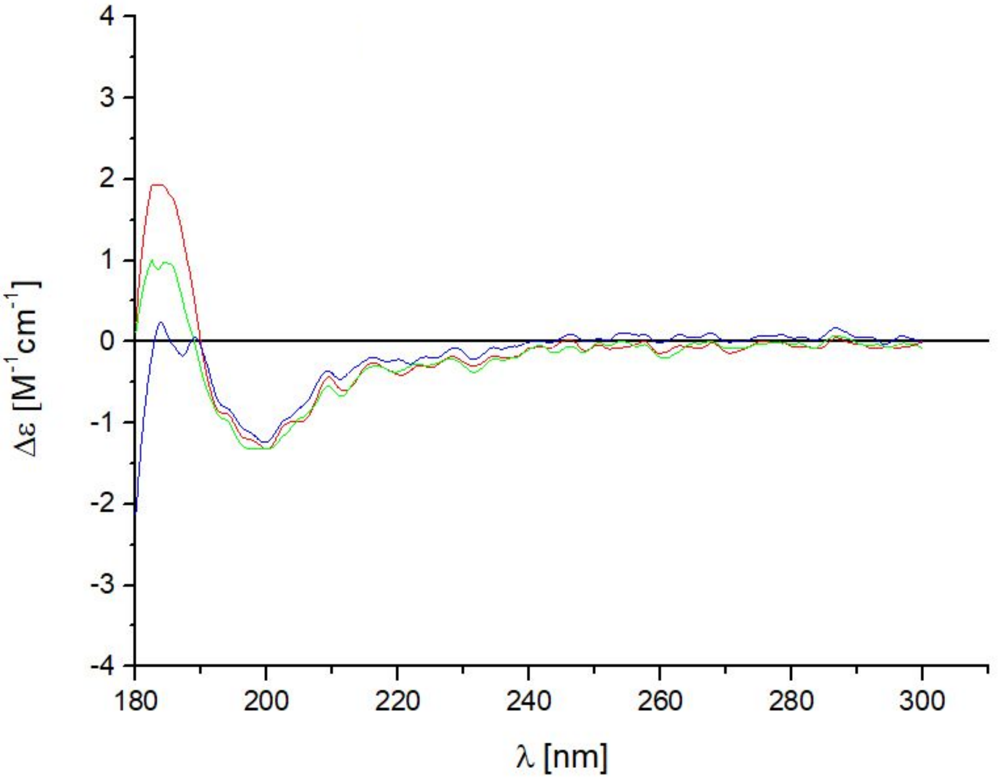

Fig. S12. CD spectrum at nm 180-320 range of Ni(II)-ligand system composed of Ni(II) ions, and Ac-$_{101}$DHHLAHIVVDAIAHASEDRR$_{120}$ peptide (L1) at pH: 5 (red), 7 (green), 10 (blue).
